# SLC25A21 downregulation promotes *KRAS*-mutant colorectal cancer progression by increasing glutamine anaplerosis

**DOI:** 10.1172/jci.insight.167874

**Published:** 2023-11-08

**Authors:** Sha-Sha Hu, Yue Han, Tian-Yuan Tan, Hui Chen, Jia-Wen Gao, Lan Wang, Min-Hui Yang, Li Zhao, Yi-Qing Wang, Yan-Qing Ding, Shuang Wang

**Affiliations:** 1 Department of Pathology, Nanfang Hospital, and; 2 Department of Pathology, School of Basic Medical Sciences, Southern Medical University, Guangzhou, China.

**Keywords:** Gastroenterology, Metabolism, Amino acid metabolism, Colorectal cancer, Drug therapy

## Abstract

Emerging evidence shows that *KRAS*-mutant colorectal cancer (CRC) depends on glutamine (Gln) for survival and progression, indicating that targeting Gln metabolism may be a promising therapeutic strategy for *KRAS*-mutant CRC. However, the precise mechanism by which Gln metabolism reprogramming promotes and coordinates *KRAS*-mutant CRC progression remains to be fully investigated. Here, we discovered that solute carrier 25 member 21 (SLC25A21) expression was downregulated in *KRAS*-mutant CRC, and that *SLC25A21* downregulation was correlated with poor survival of *KRAS*-mutant CRC patients. SLC25A21 depletion selectively accelerated the growth, invasion, migration, and metastasis of *KRAS*-mutant CRC cells in vitro and in vivo, and inhibited Gln-derived α-ketoglutarate (α-KG) efflux from mitochondria, thereby potentiating Gln replenishment, accompanied by increased GTP availability for persistent KRAS activation in *KRAS*-mutant CRC. The restoration of SLC25A21 expression impaired the *KRAS*-mutation-mediated resistance to cetuximab in *KRAS*-mutant CRC. Moreover, the arrested α-KG efflux that occurred in response to SLC25A21 depletion inhibited the activity of α-KG–dependent DNA demethylases, resulting in a further decrease in *SLC25A21* expression. Our studies demonstrate that SLC25A21 plays a significant role as a tumor suppressor in *KRAS*-mutant CRC by antagonizing Gln-dependent anaplerosis to limit GTP availability for KRAS activation, which suggests potential alternative therapeutic strategies for *KRAS*-mutant CRC.

## Graphical abstract

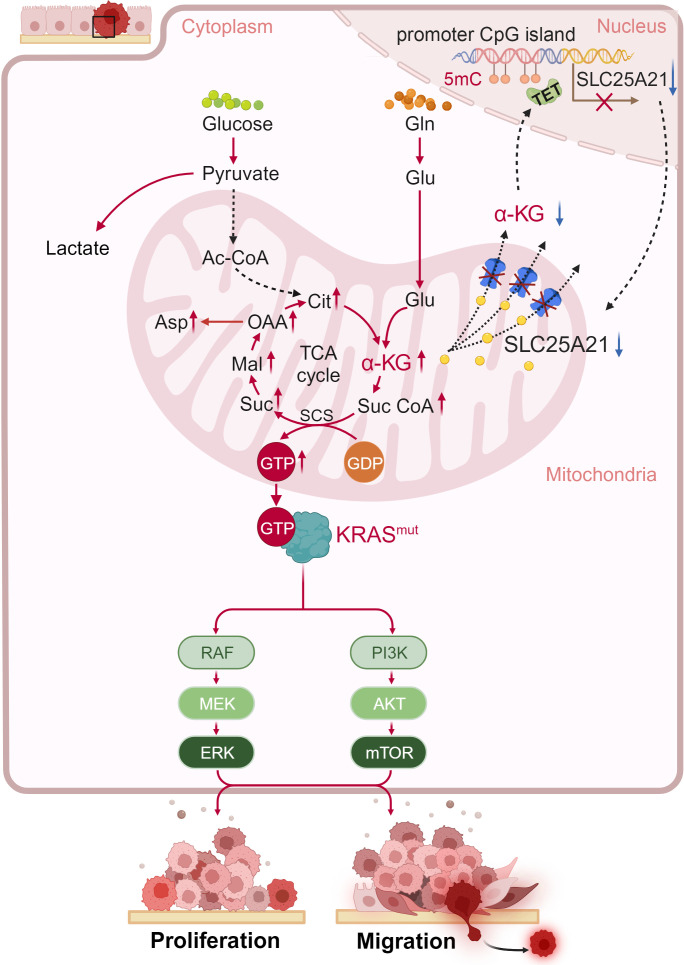

## Introduction

Colorectal cancer (CRC) is the third most common type of cancer and the second leading cause of cancer-related mortality worldwide (1). The main cause of CRC-related mortality is metastasis to distant organs, including the liver and lungs (2). Despite therapeutic advances, the 5-year survival rate of patients with metastatic CRC (mCRC) remains under 10% (3). Inhibitors targeting epidermal growth factor receptor (EGFR) have been shown to be effective for the treatment of mCRC (4); however, the efficacy of anti-EGFR therapy is often limited by intrinsic drug resistance resulting from downstream *KRAS* mutation (5, 6). Unfortunately, approximately 45% of CRC patients harbor activating *KRAS* mutations (6). Although the recently developed agent sotorasib is the first KRAS^G12C^-specific inhibitor approved for clinical use in lung cancers (7), more common targets in CRC, such as KRAS^G12D^ and KRAS^G12V^, remain “undruggable” to date. Therefore, there is an urgent need to explore and develop efficient therapeutic strategies for late-stage *KRAS*-mutant CRC.

Metabolic reprogramming is being increasingly considered a hallmark of cancer (8). Growing evidence shows that *KRAS*-mutation-driven cancer cells, including CRC, exhibit the metabolic vulnerability of being addicted to glutamine (Gln) (9–12), indicating that targeting Gln metabolism may be a promising therapeutic strategy for *KRAS*-mutation-driven cancers (13). Notably, the consensus molecular subtype 3 (CMS3) of CRC, which is enriched for *KRAS* mutations, is characterized by profound alterations in multiple metabolic signatures, including Gln metabolism (14). Although substantial research focused on metabolic changes has improved the understanding of *KRAS*-mutation-driven cancers, the precise mechanism by which metabolic reprogramming promotes and coordinates *KRAS*-mutant CRC growth and progression remains to be fully investigated.

It is increasingly appreciated that cancer cells can promote growth and dissemination by increasing the ability of cells to acquire the necessary metabolic substrates and actively changing the way nutrients are used (15). The solute carrier (SLC) superfamily plays important roles in transporting metabolites, nucleotides, coenzymes, and drugs across biological membranes (16). Recent studies have highlighted the important roles of SLCs in human cancers by demonstrating that they increase the transport of metabolites (12, 17–19). Specifically, the glutamate (Glu) transporter SLC 25 member 22 (SLC25A22) and the amino acid transporter SLC7A5 are required for the growth and survival of *KRAS*-mutant CRC (12, 18). Given that there are likely still numerous unexplored transporter alterations in CRC harboring *KRAS* mutation, we analyzed the transcript abundance of the transporters of glutaminolysis-associated metabolites in *KRAS*-mutant CRC tissues using publicly available data sets from The Cancer Genome Atlas (TCGA). Among such transporters, *SLC25A21* was identified as one of the top genes with aberrant expression. SLC25A21, an oxodicarboxylate carrier, is responsible for the transport of 2-oxoadipate (2-OA) and α-ketoglutarate (α-KG) across the inner mitochondrial membrane (IMM) (20). One study preliminarily found that *SLC25A21* expression is aberrant in nasopharyngeal carcinoma cell lines (21). Recently, we preliminarily showed that SLC25A21 suppresses cell growth by inducing oxidative stress in bladder cancer (22). However, whether SLC25A21 is responsible for rewiring Gln metabolism and how it modulates *KRAS*-mutant CRC progression are largely unknown.

In the present study, we discovered that SLC25A21 expression was downregulated in *KRAS*-mutant CRC and that *SLC25A21* downregulation was closely correlated with poor survival of *KRAS*-mutant CRC patients and selectively accelerated the cell growth, invasion, migration, and metastasis of CRC cells with *KRAS* mutation in vitro and in vivo. Mechanistic investigations revealed that SLC25A21 downregulation inhibited Gln-derived α-KG efflux and potentiated glutaminolysis to replenish the tricarboxylic acid (TCA) cycle, accompanied by an increase in GTP availability for persistent KRAS activation in *KRAS*-mutant CRC. Moreover, SLC25A21 overexpression abrogated mutant-*KRAS*-mediated resistance to cetuximab (CTX) in CRC in vitro. Altogether, our study provides insights into the significant role of SLC25A21 in *KRAS*-mutant CRC and the relevant mechanisms, and these findings may contribute to the development of new therapeutic strategies for *KRAS*-mutation-driven CRC.

## Results

### Aberrant expression of glutaminolysis-associated transporters is a common feature in KRAS-mutant CRC.

Although it is known that glutaminolysis, the sequence of enzymatic reactions, is effective for energy production and biosynthesis, the functions of transporters of glutaminolysis metabolites are largely unknown. Thus, we first analyzed the transcriptional levels of 29 SLC transporters of glutaminolysis-associated metabolites in human *KRAS*-mutant CRC and adjacent normal samples from TCGA and found 21 transporters with significantly differential expression, including 8 upregulated and 13 downregulated ones in *KRAS*-mutant CRC (*P* < 0.05, Supplemental Figure 1; supplemental material available online with this article; https://doi.org/10.1172/jci.insight.167874DS1). Because mitochondrial metabolism is essential for *KRAS*-mediated tumorigenesis (23), we paid close attention to mitochondrial SLCs. *SLC25A22* was the only upregulated gene in *KRAS*-mutant CRC samples and has been demonstrated to promote *KRAS*-mutant CRC cell survival by increasing Glu influx from the cytosol into the mitochondria (12). Among the downregulated SLCs, *SLC25A21* was the transporter with the most differential expression. SLC25A21 carriers catalyze the transport of 2-OA and α-KG by a counter-exchange mechanism (20). Given that α-KG is an essential metabolite in glutaminolysis (24), we focused on *SLC25A21* and investigated its effects on and the mechanisms by which it rewires Gln metabolism in *KRAS*-mutant CRC.

### SLC25A21 expression is downregulated in CRC and is positively correlated with prognosis in KRAS-mutant CRC.

**Figure 1:**
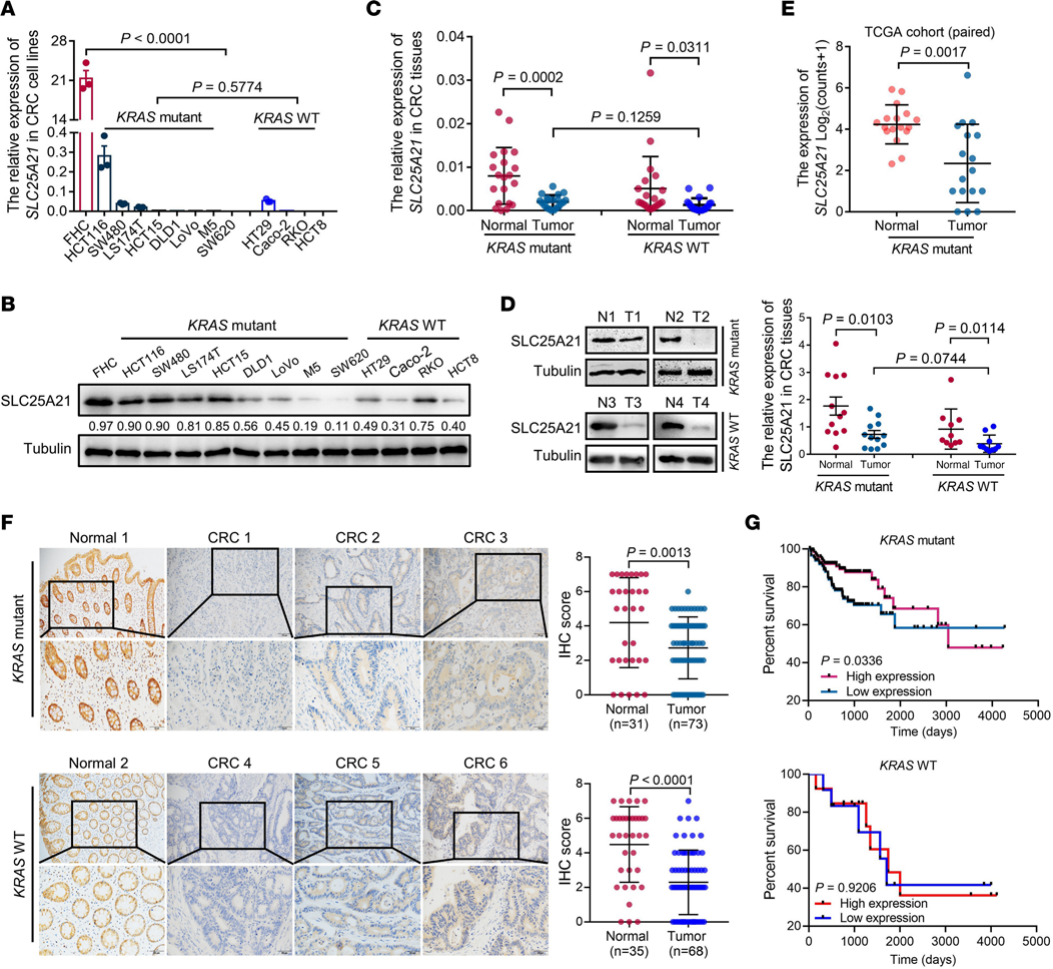
SLC25A21 expression is downregulated in CRC and positively correlated with prognosis in patients with *KRAS*-mutant CRC. (**A**) The *SLC25A21* transcript levels in an immortalized colon mucosa epithelial cell line (FHC) and CRC cell lines were quantified by real-time RT-PCR (*n* = 3 biologically independent experiments). (**B**) Immunoblot analysis of SLC25A21 protein in FHC and CRC cells from **A**. An anti-tubulin antibody was used for normalization. (**C** and **D**) SLC25A21 mRNA (**C**, *n* = 20 per group) and protein levels (**D**, *KRAS* mutation, *n* = 12; WT *KRAS*, *n* = 10) in paired CRC and adjacent noncancerous tissues. (**E**) Transcript abundance of *SLC25A21* in paired *KRAS*-mutant CRC and noncancerous tissue samples from TCGA (*n* = 17). (**F**) Representative micrographs of SLC25A21 expression patterns in normal colorectal mucosa and CRC tissues by IHC (left). Scale bars: 100 μm (top) or 50 μm (bottom). Quantification of SLC25A21 IHC staining in normal colorectal mucosa and CRC tissues (right upper, *KRAS* mutation, normal, *n* = 31; tumor, *n* = 73. right bottom, WT *KRAS*, normal, *n* = 35; tumor, *n* = 68). (**G**) Kaplan-Meier survival curves for CRC patients with *KRAS* mutation (upper, high expression, *n* = 117; low expression, *n* = 114) and WT *KRAS* (bottom, high expression, *n* = 12; low expression, *n* = 13) stratified by the median level of *SLC25A21* expression from TCGA. The immunoblots in **B** are representative of 2 independent experiments. Data are represented as mean ± SD. Statistical significance was calculated by unpaired, 2-sided *t* test (**A**, **C**, **D**, and **F**), paired, 2-sided *t* test (**C**–**E**), 1-way ANOVA with Dunnett’s post hoc test (**A**, comparison between FHC cells and CRC cell lines), and log-rank test (**G**); the *P* values are shown.

We next assessed the endogenous expression levels of *SLC25A21* in a panel of cell lines and in paired CRC and adjacent noncancerous mucosa tissue samples. Compared with that in FHC cells, *SLC25A21* was significantly downregulated in *KRAS*-mutant CRC cell lines (*P* < 0.0001, Figure 1A). A similar trend was observed in CRC cells with wild-type (WT) *KRAS*, but no significant differences were found between the 2 groups of CRC cell lines (*P* = 0.5774, Figure 1A). These findings were also reflected at the protein level (Figure 1B). Consistent with the cell line data, the SLC25A21 mRNA (*P* = 0.0002, Figure 1C) and protein (*P* = 0.0103, Figure 1D) were lower in *KRAS*-mutant CRC tissues than in paired noncancerous tissues. Correspondingly, downregulated expression was also observed in CRC tissues with WT *KRAS* (*P* < 0.05), but we did not find significant differences between the 2 groups of CRC tissues with *KRAS* mutation and WT *KRAS* (Figure 1, C and D). Moreover, the *SLC25A21* downregulation was found in *KRAS*-mutant CRC tissues compared with paired normal samples from TCGA cohort (*P* = 0.0017, Figure 1E). Furthermore, IHC assays revealed that SLC25A21 protein expression was heterogeneous and confirmed a significant decrease in the SLC25A21 protein level in both *KRAS*-mutant and *KRAS*-WT CRC tissues (*P* < 0.01, Figure 1F), but no significant differences were found between the 2 groups of CRC tissues (*P* = 0.1641).

We then validated the prognostic significance of *SLC25A21* levels in CRC patients using TCGA data. Interestingly, *SLC25A21* downregulation was correlated with poor survival in *KRAS*-mutant CRC (log-rank test *P* = 0.0336), but not in CRC with WT *KRAS* (log-rank test *P* = 0.9206, Figure 1G).

### SLC25A21 selectively inhibits the growth of KRAS-mutant CRC cells in vitro.

**Figure 2:**
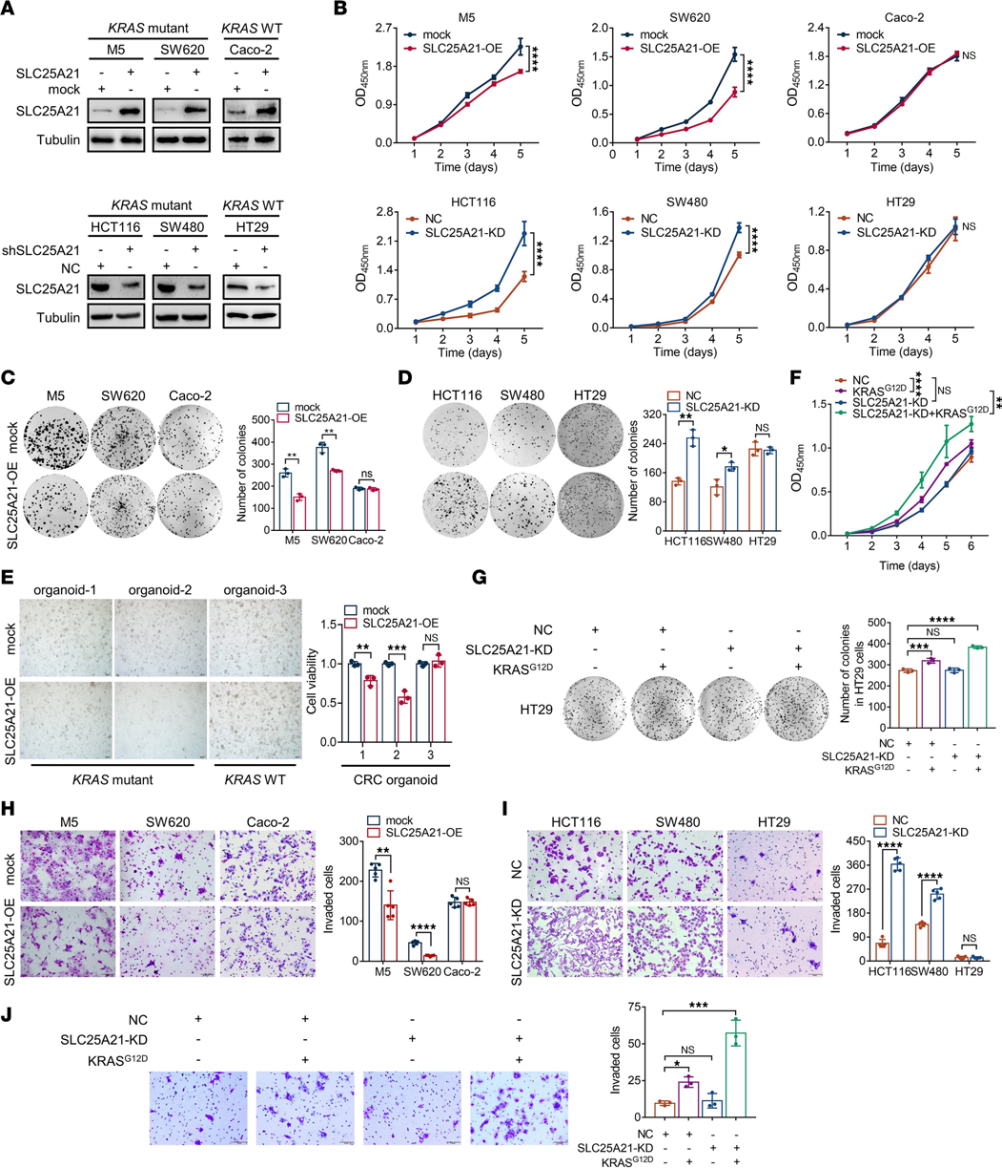
SLC25A21 inhibits cell growth and invasion in *KRAS*-mutant CRC in a *KRAS*-mutation-dependent manner in vitro. (**A**) Immunoblot analysis of SLC25A21 protein levels in CRC cells with or without SLC25A21 overexpression or knockdown. (**B**) Proliferation of *KRAS*-mutant and *KRAS*-WT CRC cells with SLC25A21 overexpression (upper, *n* = 3 biologically independent experiments) or knockdown (bottom, *n* = 3–5 biologically independent experiments). (**C** and **D**) Representative images (left) and quantification (right) showing the colony-forming capacity of *KRAS*-mutant and *KRAS*-WT CRC cells with SLC25A21 overexpression (**C**, *n* = 3 biologically independent experiments) or silencing (**D**, *n* = 3 biologically independent experiments). (**E**) Representative images (left) and quantification (right) showing the growth of primary human CRC organoids with *KRAS* mutation or WT *KRAS*. (**F** and **G**) Proliferation (**F**, *n* = 3 biologically independent experiments) and colony-forming capacities (**G**, *n* = 3 biologically independent experiments) of HT29 cells with or without expression of mutated KRAS^G12D^ or SLC25A21 knockdown. (**H** and **I**) Invasiveness of *KRAS*-mutant and *KRAS*-WT CRC cells with SLC25A21 overexpression (**H**) or knockdown (**I**, *n* = 5 biologically independent experiments). (**J**) Invasiveness of HT29 cells from **G**, treated as shown (*n* = 3 biologically independent experiments). Scale bars: 50 μm. SLC25A21-OE, SLC25A21 overexpression; SLC25A21-KD, SLC25A21 knockdown; NS, nonsignificant. The immunoblots in **A** are representative of 2 independent experiments. Data are presented as the mean ± SD. Statistical significance was calculated by 2-way ANOVA with Bonferroni’s post hoc test (**B** and **F**), unpaired, 2-sided *t* test (**C**–**E**, **H**, and **I**), and 1-way ANOVA with Dunnett’s post hoc test (**G** and **J**). *P < 0.05; **P < 0.01; ***P < 0.001; ****P < 0.0001.

To determine the functional consequence of SLC25A21 downregulation in *KRAS*-mutant CRC cells, 2 *KRAS*-mutant CRC cell lines (M5 and SW620) and 1 *KRAS*-WT cell line (Caco-2), which have relatively low endogenous SLC25A21 expression, were used to establish stable SLC25A21-overexpressing cells upon transfection with the LV5-*SLC25A21* lentiviral vector. Moreover, we knocked down *SLC25A21* expression in *KRAS*-mutant HCT116 and SW480 cells and in *KRAS*-WT HT29 cells, which have relatively high endogenous expression, by infection with a lentiviral vector harboring shRNA-*SLC25A21*. The efficiency of overexpression and silencing was verified in the corresponding CRC cells at both the transcript and protein levels (Supplemental Figure 2A, Supplemental Figure 2B, and Figure 2A). SLC25A21 overexpression significantly suppressed the proliferation rate and colony-forming capacity of *KRAS*-mutant CRC cells, M5 and SW620 (*P* < 0.01), but had a minimal effect on Caco-2 cells, a *KRAS*-WT CRC cell line (*P* > 0.05, Figure 2B and Figure 2C). In contrast, *SLC25A21* silencing led to accelerated proliferation and increased colony formation in *KRAS*-mutant CRC cells, but not in *KRAS*-WT HT29 cells (Figure 2B and Figure 2D). To further assess the growth suppression mediated by SLC25A21, we transfected the LV5-*SLC25A21* vector into 3 human primary CRC organoids and found that SLC25A21 overexpression suppressed organoid growth in Matrigel from CRC with *KRAS* mutation; however, SLC25A21 overexpression in *KRAS*-WT CRC organoids did not alter their growth in vitro (Figure 2E).

To determine whether a direct causal link exists between the acquisition of *KRAS* mutation and the dysregulation of SLC25A21 expression, we introduced KRAS^G12D^ into HT29 cells. The introduction of KRAS^G12D^ into HT29 cells did not alter SLC25A21 expression (Supplemental Figure 2C). Interestingly, SLC25A21 depletion increased the proliferation rate and colony formation capacity of HT29 cells in a *KRAS*-mutation-dependent manner (Figure 2, F and G). In addition, we determined the effects of SLC25A21 on pancreatic ductal adenocarcinoma (PDAC) cells with *KRAS* mutation (AsPC-1 and MIA PaCa-2) or WT *KRAS* (BxPC-3) and found that SLC25A21 upregulation inhibited the proliferation and colony formation of the 2 *KRAS*-mutant PDAC cell lines, but not of BxPC-3 cells (Supplemental Figure 3). Together, these data indicate that SLC25A21 selectively affects *KRAS*-mutant cancer cells.

### SLC25A21 selectively suppresses cell invasion and migration in KRAS-mutant CRC cells in vitro.

We conducted Matrigel invasion and wound-healing assays to assess the effects of SLC25A21 on cell invasion and migration. The invasion assays illustrated that ectopic overexpression of SLC25A21 markedly suppressed cell invasion in *KRAS*-mutant CRC cells (M5 cells, *P* = 0.0012; SW620 cells, *P* < 0.0001) but not in Caco-2 cells (*P* = 0.9546, Figure 2H). Moreover, wound-healing assays revealed that SLC25A21 overexpression significantly reduced the migration potential of both M5 and SW620 cells (*P* < 0.01), but not of Caco-2 cells (*P* = 0.6360, Supplemental Figure 4A). In contrast, SLC25A21 downregulation selectively promoted cell invasion (*P* < 0.0001) and migration (*P* < 0.05) in *KRAS*-mutant cells (Figure 2I and Supplemental Figure 4B). Similarly, SLC25A21 depletion also increased the migration and invasion abilities of HT29 cells expressing KRAS^G12D^ (Figure 2J and Supplemental Figure 4C). Hence, SLC25A21 plays important suppressor roles in the cell migration and invasion of *KRAS*-mutant CRC cells.

### SLC25A21 inhibits tumorigenicity and metastasis of KRAS-mutant CRC cells in vivo.

**Figure 3:**
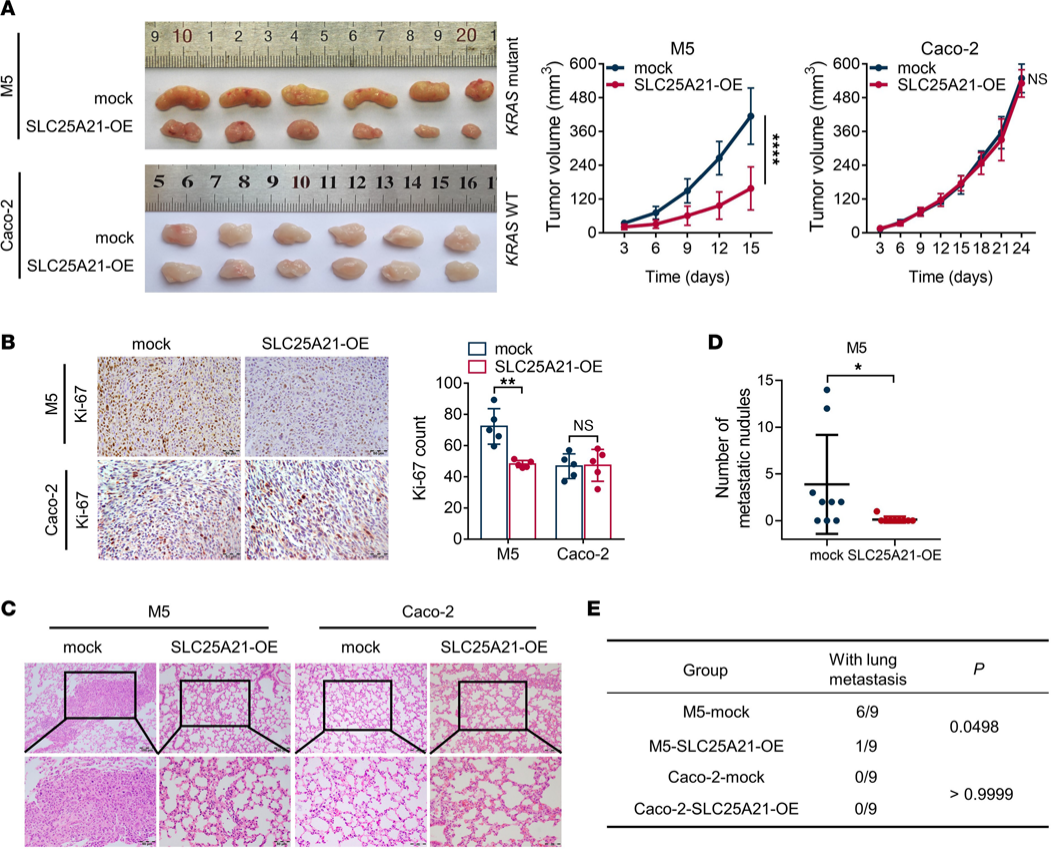
SLC25A21 inhibits the tumorigenicity and metastasis of *KRAS*-mutant CRC cells in vivo. (**A**) Bright-field images of tumors (left) and growth curves of tumor volume (right) from nude mice ectopically transplanted with CRC cells with or without SLC25A21 overexpression (*n* = 6 per group). The graphs show data from the tumor xenografts at 15 or 24 days after cells were ectopically and subcutaneously implanted. (**B**) Representative IHC staining (left) and quantification (right) showing the Ki-67 index of tumor xenografts from CRC cells (*n* = 5). Scale bars: 50 μm. (**C**) Representative H&E staining images showing lung metastases from nude mice 4 weeks after tail vein injection of CRC cells with or without SLC25A21 overexpression (*n* = 9 per group). Scale bars: 100 μm (top) or 50 μm (bottom). (**D**) Quantification of pulmonary tumor colonies after tail vein injection of CRC cells with SLC25A21 overexpression or control cells. (**E**) Statistical comparisons of lung metastases after tail vein injection of SLC25A21-overexpressing and control cells (*n* = 9 per group). Data are presented as the mean ± SD. Statistical significance was calculated by 2-way ANOVA with Bonferroni’s post hoc test (**A**), unpaired 2-sided *t* test (**B** and **D**), and Fisher’s exact test (**E**). *P < 0.05; **P < 0.01; ****P < 0.0001.

To further confirm our in vitro findings, we evaluated the effects of SLC25A21 on xenograft tumor growth in vivo. SLC25A21-overexpressing M5 and Caco-2 cells, SLC25A21-depleted HCT116 cells, and the corresponding control cells were implanted into nude mice. As shown in Figure 3A, SLC25A21 overexpression severely arrested tumor growth in mice injected with M5 cells, whereas tumor growth continued unabated in mice injected with control cells (*P* *<* 0.0001, Figure 3A). However, SLC25A21 overexpression did not exert a significant effect on Caco-2 xenograft growth (*P* *>* 0.05, Figure 3A). SLC25A21 overexpression significantly decreased the Ki-67 index in M5 cell xenografts but not in Caco-2 cells, as shown by IHC (Figure 3B). In contrast, the knockdown of SLC25A21 expression in HCT116 cells significantly accelerated xenograft tumor growth in nude mice and increased the Ki-67 index in xenografts (Supplemental Figure 5, A and B).

We further explored the role of SLC25A21 in lung metastasis by establishing a tail vein injection model in nude mice. The results showed that SLC25A21 upregulation markedly decreased the in vivo metastasis of CRC cells. M5 cells overexpressing SLC25A21 formed lung metastases in only 1 out of 9 mice, whereas lung metastasis was identified in 6 out of 9 control mice. However, in *KRAS*-WT CRC cells, neither SLC25A21-overexpressing Caco-2 nor control cells showed lung metastasis (Figure 3, C–E). In addition, *SLC25A21* silencing increased the lung metastasis of HCT116 cells with *KRAS* mutation (Supplemental Figure 5, C–E). Collectively, the in vivo data indicate that SLC25A21 plays an important inhibitory role in tumor growth and metastasis, consistent with the results from in vitro assays.

### SLC25A21 downregulation promotes glutaminolysis by restricting Gln-derived α-KG efflux to replenish metabolites needed for the TCA cycle.

**Figure 4:**
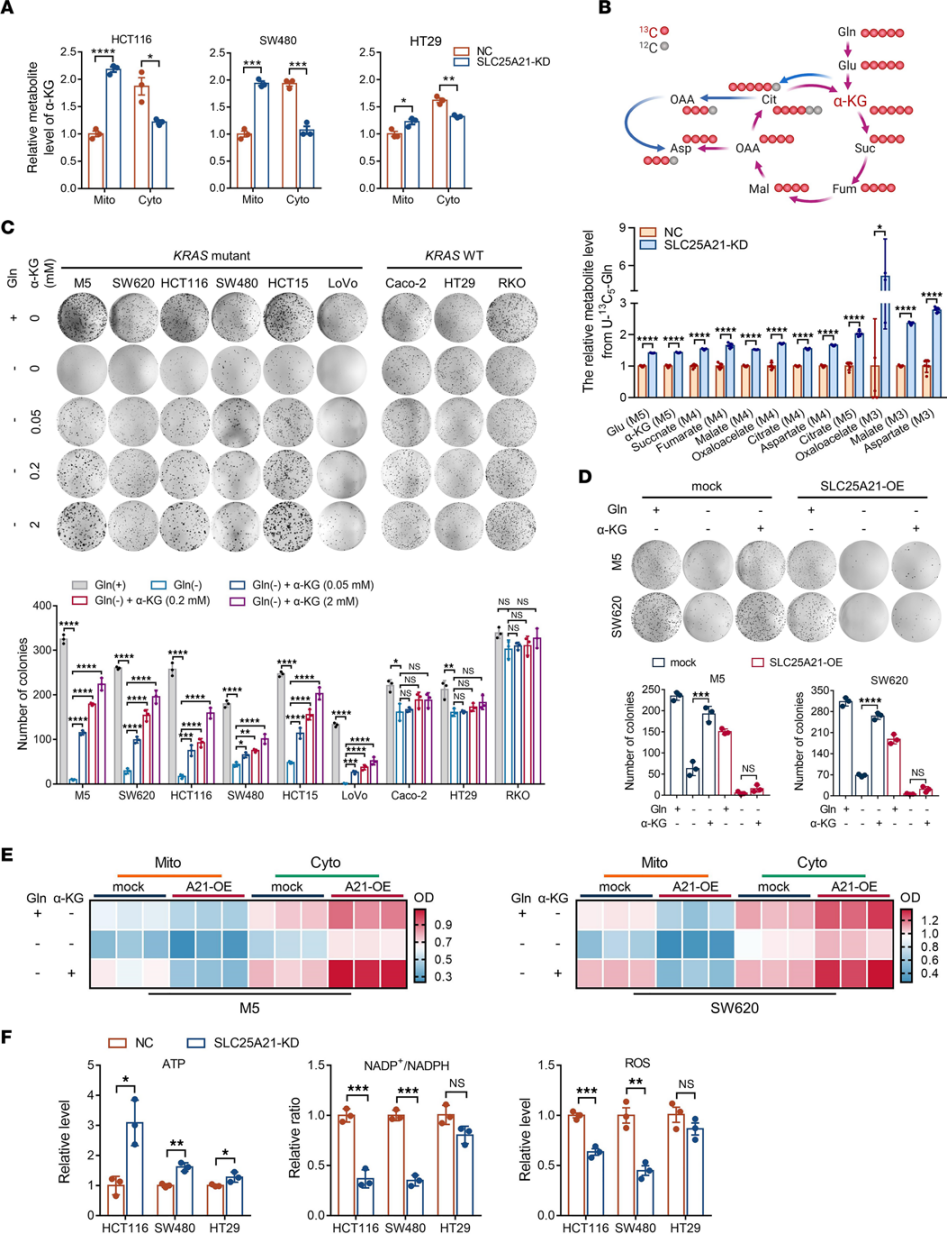
SLC25A21 downregulation promotes glutaminolysis by restricting Gln-derived α-KG efflux to replenish the TCA cycle in *KRAS*-mutant CRC cells. (**A**) Relative subcellular α-KG levels in CRC cells with and without SLC25A21 knockdown (*n* = 3 biologically independent experiments). (**B**) Diagram of Gln catabolic pathway using [U-^13^C^5^]Gln as a tracer (red) (upper) and relative abundances of labeled intracellular metabolites in HCT116 cells determined by LC-MS (bottom; *n* = 5 biologically independent samples). (**C**) Representative images (upper) and quantification (bottom) showing the colony-forming capacity of CRC cells with or without *KRAS* mutation cultured in medium under Gln-containing, Gln-free, and various α-KG–supplemented conditioned medium, treated as shown (*n* = 3 biologically independent experiments). Gln+, full medium containing Gln (2 mM); Gln–, Gln-free medium; Gln– + α-KG, Gln-free medium with various contents of dm-KG (cell-permeable analog of α-KG). (**D**) Representative images (upper) and quantification (bottom) of the colony-forming capacity of *KRAS*-mutant CRC cells with or without SLC25A21 overexpression cultured under the indicated condition, treated as shown (*n* = 3 biologically independent experiments). (**E**) Heatmap showing the relative levels of subcellular α-KG in *KRAS*-mutant CRC cells from **D**, treated as shown (*n* = 3 biologically independent experiments). Gln+, full medium containing Gln (Gln 2 mM); α-KG+, 2 mM α-KG; Gln–, Gln-free medium; A21-OE, SLC25A21 overexpression. (**F**) Relative levels of ATP and ROS and relative ratio of NADP^+^/NADPH in CRC cells with or without SLC25A21 knockdown (*n* = 3 biologically independent experiments). Data are presented as the mean ± SD. Statistical significance was calculated by unpaired, 2-sided *t* test (**A**, **B**, **D**, and **F**) and 1-way ANOVA with Tukey‘s post hoc test (**C**). *P < 0.05; **P < 0.01; ***P < 0.001; ****P < 0.0001.

We next dissected the molecular mechanisms by which SLC25A21 affects *KRAS*-mutant CRC. As predicted, in both *KRAS*-mutant and *KRAS*-WT CRC cells, SLC25A21 knockdown led to an accumulation of α-KG in mitochondria (*P* < 0.05); however, SLC25A21 depletion only increased the mitochondrial α-KG content by 22% in *KRAS*-WT cells (Figure 4A). Given the notion that α-KG is an important intermediate metabolite of glutaminolysis, we wondered whether SLC25A21 affects Gln metabolism in *KRAS*-mutant CRC. Therefore, we performed a targeted metabolomics analysis using [U-^13^C_5_]Gln as a tracer to monitor the fate of Gln in HCT116 cells by liquid chromatography–mass spectrometry (LC-MS). The results showed that SLC25A21 knockdown led to increased Glu (m+5) and α-KG (m+5) levels. Interestingly, we also observed significant increases in succinate (m+4), fumarate (m+4), malate (m+4), oxaloacetate (m+4), and citrate (m+4), indicating extensive incorporation of Gln into the TCA cycle (Figure 4B). In line with the enhanced incorporation of Gln-derived α-KG into the downstream intermediates of the TCA cycle, SLC25A21 downregulation led to decreased α-KG/succinate, α-KG/fumarate, and α-KG/malate ratios (*P* *<* 0.05, Supplemental Figure 6A). In addition, relative enhancement of reductive metabolism, as indicated by increases in citrate (m+5), was observed after SLC25A21 depletion (*P* < 0.0001, Figure 4B). Collectively, these data suggests that SLC25A21 may play a role in the maintenance of Gln-derived α-KG that participates in increased oxidative and reductive metabolism in mitochondria indicating that SLC25A21 depletion facilitates glutaminolysis in *KRAS*-mutant CRC cells. Furthermore, in addition to the increases in TCA cycle intermediates, the levels of aspartate (m+4) and aspartate (m+3), which are generated from oxaloacetate (m+4) and oxaloacetate (m+3), respectively, were also increased (*P* < 0.05, Figure 4B). In summary, these findings indicate that SLC25A21 downregulation drives energy and biosynthesis by allowing the continuous replenishment of Gln-derived α-KG into the TCA cycle.

We next investigated whether the effect of Gln on *KRAS*-mutant CRC cells depends on α-KG maintenance in mitochondria mediated by SLC25A21 downregulation. We performed Gln deprivation and α-KG rescue assays and found that *KRAS*-mutant CRC cells were sensitive to Gln deprivation, whereas *KRAS*-WT CRC cells were relatively tolerant of Gln deprivation (Figure 4C). When Gln was removed from the culture medium, the addition of dimethyl-α-KG (dm-KG), a cell-permeable analog of α-KG, supported the growth of *KRAS*-mutant CRC cells but not *KRAS*-WT cells (Figure 4C). To assess the role of SLC25A21 in *KRAS*-mutant CRC cells with rewired Gln metabolism, we increased SLC25A21 expression to accelerate α-KG efflux. As shown in Figure 4, D and E, assays of the subcellular distribution of α-KG and colony formation revealed that Gln could maintain the α-KG content in mitochondria to support cell growth under Gln-repleted conditions, but SLC25A21 overexpression induced mitochondrial α-KG efflux and significantly decreased the colony-forming capacity of *KRAS*-mutant cells, as previously observed (Figure 2C). Intriguingly, the addition of α-KG to the culture medium restored the level of mitochondrial α-KG and profoundly rescued the previously observed growth defects mediated by Gln deprivation in control cells. However, under Gln-deprived conditions, SLC25A21 overexpression induced a significant decrease in α-KG content in mitochondria, even with the addition of α-KG (Figure 4E), and this addition failed to restore the proliferation defects of SLC25A21-overexpressing cells (*P* > 0.05, Figure 4D). These data imply that SLC25A21 depletion, which arrests mitochondrial α-KG efflux, is needed for Gln to fuel proliferation in *KRAS*-mutant CRC.

Moreover, a marked increase in ATP production was observed as a result of SLC25A21 downregulation in *KRAS*-mutant cells. In *KRAS*-WT cells, SLC25A21 downregulation increased ATP production, but this effect was less substantial than that observed in *KRAS*-mutant cells. In addition, SLC25A21 depletion decreased the NADP^+^/NADPH ratio and reactive oxygen species (ROS) production in *KRAS*-mutant CRC cells (Figure 4F), indicating enhanced redox homeostasis. Furthermore, we suppressed the level of oxidative phosphorylation (OxPHOS) in CRC cells with IACS-010759 (100 nM), an inhibitor of OxPHOS, to test the contribution of OxPHOS to the important role of SLC25A21 depletion. In *KRAS*-mutant CRC cells, IACS-010759 treatment could restore the promoting effect mediated by SLC25A21 depletion on colony formation to a level similar to that found with the control cells. However, neither SLC25A21 depletion nor IACS-010759 treatment had a significant effect on the growth of *KRAS*-WT cells (Supplemental Figure 6B). These data indicate that SLC25A21-depleted *KRAS*-mutant CRC cells are more sensitive to OxPHOS inhibitors.

### SLC25A21 downregulation promotes KRAS activity and activates the downstream PI3K/AKT and RAF/ERK signaling pathways.

**Figure 5:**
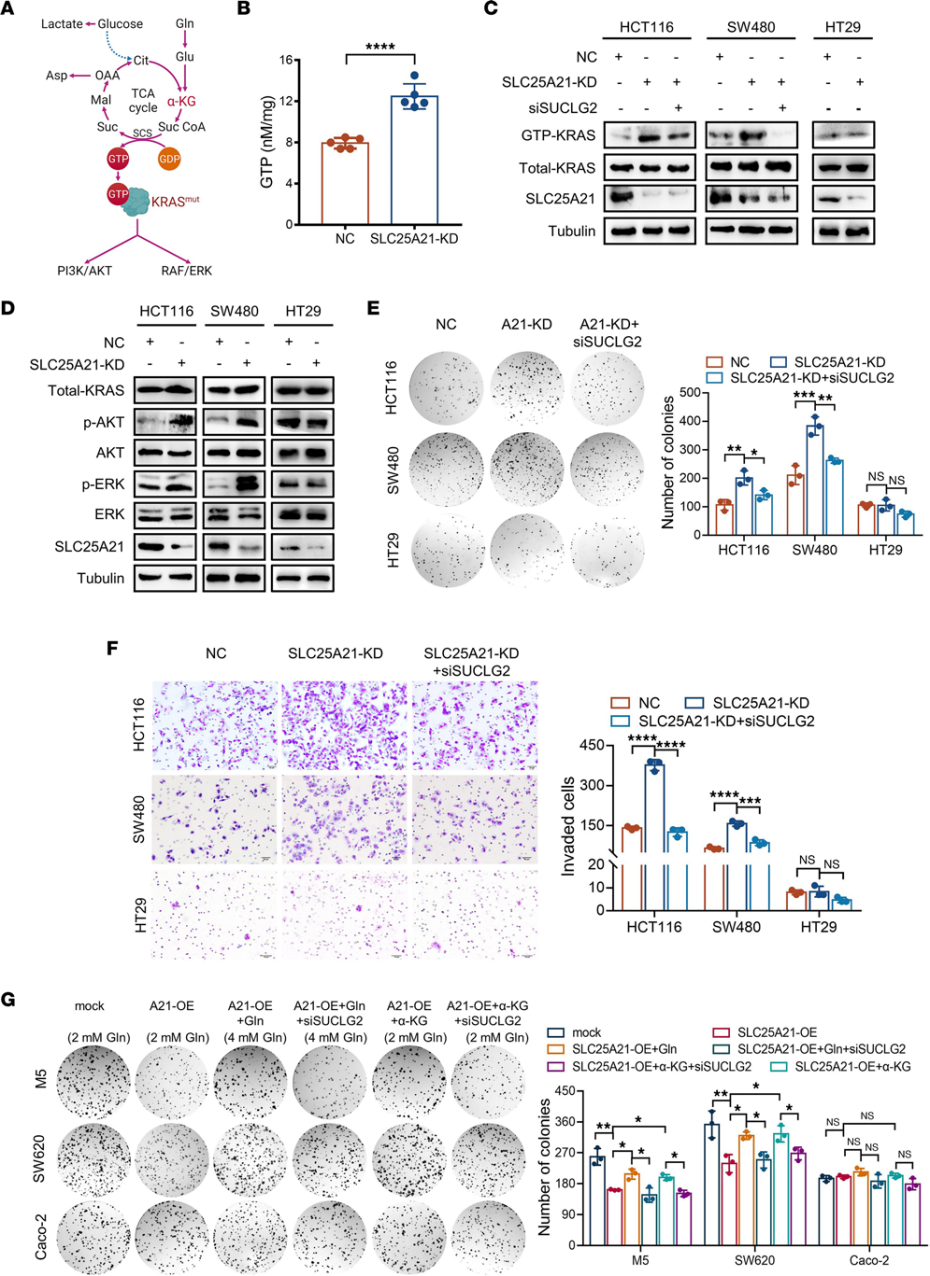
The ability of SLC25A21 downregulation to promote KRAS activity and cell proliferation requires SUCLG2 in *KRAS*-mutant CRC cells. (**A**) Schematic diagram of the effect of glutaminolysis on KRAS-mediated downstream signaling pathways. (**B**) Quantitative analyses of the intracellular GTP levels by LC-MS/MS (*n* = 5 biologically independent samples). (**C**) Immunoblot analysis of KRAS activity in *KRAS*-mutant and *KRAS*-WT CRC cells with or without SLC25A21 or SUCLG2 depletion. (**D**) Immunoblot analysis of the activity of the PI3K/AKT and RAF/ERK pathways in *KRAS*-mutant and *KRAS*-WT CRC cells with or without SLC25A21 knockdown. (**E** and **F**) Representative images (left) and quantification (right) of the colony-forming capacity (**E**) and invasiveness (**F**) of CRC cells from **C**, treated as shown (*n* = 3 biologically independent experiments). Scale bars: 50 μm. (**G**) Representative images (left) and quantification (right) of the colony-forming capacity of CRC cells with or without SLC25A21 overexpression or SUCLG2 knockdown in the absence or presence of α-KG or Gln addition, treated as shown (*n* = 3 biologically independent experiments). Cells were cultured in normal medium (Gln-containing, 2 mM) or conditioned medium with α-KG addition (2 mM) or Gln addition (total of 4 mM Gln). The immunoblots in **C** and **D** are representative of 2 independent experiments. Data are represented as mean ± SD. Statistical significance was calculated by unpaired, 2-sided *t* test (**B**) and 1-way ANOVA with Tukey‘s post hoc test (**E**–**G**). *P < 0.05; **P < 0.01; ***P < 0.001; ****P < 0.0001.

KRAS is a small GTPase that normally cycles between a GTP-bound active and a GDP-bound inactive form. KRAS mutation favors the formation of persistently GTP-bound KRAS, the active state. In the TCA cycle, α-KG undergoes oxidative decarboxylation to succinyl-CoA, and CoA from succinyl-CoA is then removed to generate free succinate via a process coupled to the substrate-level phosphorylation of GDP to GTP, the only way to directly generate a high-energy phosphate bond reaction in the TCA cycle (Figure 5A). We hypothesized that SLC25A21-depletion-mediated α-KG arrest increases GTP production and thereby promotes persistent KRAS activation in *KRAS*-mutant CRC. LC–tandem MS (LC-MS/MS) analysis showed a significantly elevated GTP abundance in SLC25A21-depleted HCT116 cells (*P* < 0.0001, Figure 5B). Accordingly, in all *KRAS*-mutant cell lines, SLC25A21 depletion increased KRAS activity, whereas SLC25A21 overexpression inhibited KRAS activity. However, SLC25A21 depletion had little effect on *KRAS*-WT CRC cells (Figure 5C and Supplemental Figure 7A).

KRAS mediates the downstream PI3K/AKT and RAF/ERK signaling pathways to promote cancer cell proliferation and metastasis (25). Hence, we sought to examine the effect of SLC25A21 on these pathways. The levels of p-AKT/p-ERK were increased and decreased in SLC25A21-depleted and SLC25A21-overexpressing CRC cells with *KRAS* mutation (Figure 5D and Supplemental Figure 7B), respectively, whereas no significant differences were found in *KRAS*-WT HT29 cells (Figure 5D). Collectively, these data indicate that SLC25A21 inhibits cancer cell growth and metastasis by repressing the KRAS/AKT/ERK pathway in *KRAS*-mutant CRC.

### The effects of SLC25A21 downregulation on KRAS-mutant CRC depend on Gln-derived α-KG–mediated replenishment of the TCA cycle.

α-KG undergoes oxidative decarboxylation to generate succinyl-CoA and subsequently succinate. Succinate-CoA ligase GDP-forming subunit-β (SUCLG2), a subunit of succinyl-CoA synthetase (SCS), is critical for the conversion of succinyl-CoA into succinate. To further ascertain the contribution of Gln-derived α-KG–mediated replenishment of the TCA cycle to the effects of SLC25A21, we inhibited the conversion of α-KG into succinate via an siRNA targeting *SUCLG2* in SLC25A21-depleted CRC cells. Remarkably, SUCLG2 downregulation completely abolished the increases in KRAS activity, colony formation, and migration mediated by SLC25A21 downregulation in *KRAS*-mutant CRC cells (Figure 5, C, E, and F, and Supplemental Figure 8A). However, SUCLG2 downregulation had little effect on *KRAS*-WT cells (Figure 5, E and F, and Supplemental Figure 8A). Moreover, in *KRAS*-mutant CRC cells, supplementation with α-KG or additional Gln in Gln-containing full medium rescued the decrease in proliferation and migration mediated by SLC25A21 overexpression, whereas SUCLG2 downregulation abolished the rescue effect of Gln or α-KG supplementation in culture medium on the proliferation and migration of SLC25A21-overexpressing cells, but not *KRAS*-WT CRC cells (Figure 5G and Supplemental Figure 8B). Thus, the rescue effects of SUCLG2 knockdown or α-KG/Gln supplementation confirmed the crucial role of SLC25A21 in repressing *KRAS*-mutant CRC progression by arresting α-KG efflux from mitochondria and thus promoting anaplerotic Gln flux into the TCA cycle.

### Restoration of SLC25A21 expression abrogates KRAS-mutation-mediated resistance to cetuximab in CRC.

**Figure 6:**
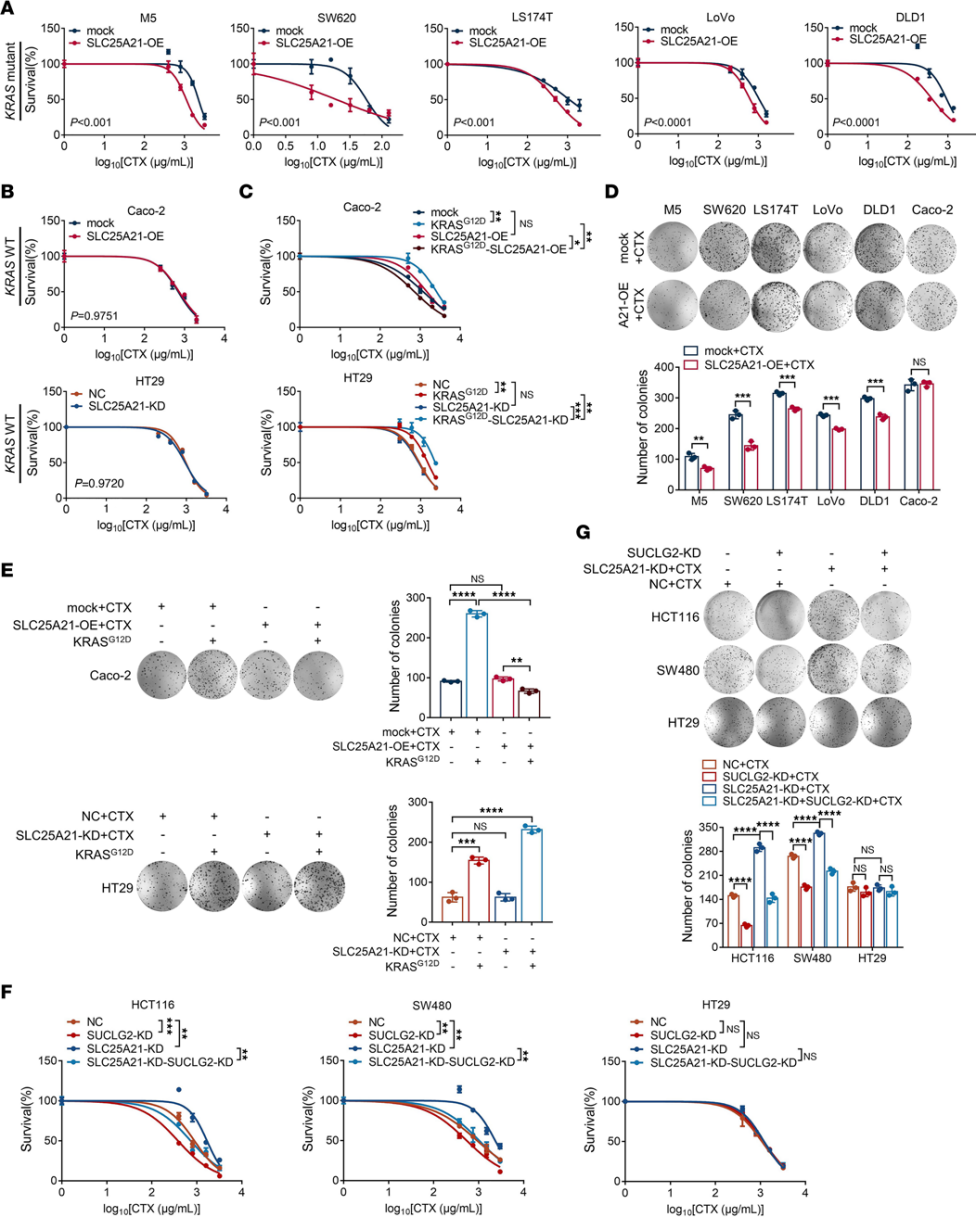
The restoration of SLC25A21 expression abrogates *KRAS*-mutation-mediated resistance to CTX in CRC. (**A**) IC_50_ curves for CTX in *KRAS*-mutant CRC cells with or without SLC25A21 overexpression (*n* = 3 biologically independent experiments). (**B**) IC_50_ curves for CTX in *KRAS*-WT CRC cells with or without SLC25A21 overexpression or SLC25A21 knockdown (*n* = 3 biologically independent experiments). (**C**) IC_50_ curves for CTX in CRC cells from **B** expressing mutated KRAS^G12D^, treated as shown (*n* = 3 biologically independent experiments). (**D**) Representative images (upper) and quantification (bottom) of the colony-forming capacity of CRC cells with or without SLC25A21 overexpression in the presence of CTX for 48 hours (*n* = 3 biologically independent experiments). (**E**) Representative images (left) and quantification (right) of the colony-forming capacity of CRC cells in the presence of CTX for 48 hours, treated as shown (*n* = 3 biologically independent experiments). (**F**) IC_50_ curves for CRC cells with or without SLC25A21 and SUCLG2 knockdown (*n* = 3 biologically independent experiments). (**G**) Representative images (left) and quantification (right) of the colony-forming capacity of CRC cells in the presence of CTX for 48 hours, treated as shown (*n* = 3 biologically independent experiments). Data are represented as mean ± SD. Statistical significance was calculated by 2-way ANOVA followed by correction for multiple comparisons (**A**–**C** and **F**), 1-way ANOVA with Tukey’s post hoc test (**E** and **G**), and unpaired, 2-sided *t* test (**D**). *P < 0.05; **P < 0.01; ***P < 0.001; ****P < 0.0001.

*KRAS* mutation, which results in hyperactive PI3K/AKT and RAF/ERK signaling (26), is responsible for resistance to anti-EGFR antibody therapy (27). After establishing the potent inhibitory effect of SLC25A21 on PI3K/AKT and RAF/ERK activity in *KRAS*-mutant CRC, we hypothesized that SLC25A21 affects the sensitivity of *KRAS*-mutant cells to anti-EGFR antibody therapy in CRC. As expected, SLC25A21 overexpression restored CTX sensitivity in 5 *KRAS*-mutant cell lines (Figure 6A). However, the manipulation of SLC25A21 levels did not affect the CTX sensitivity of *KRAS*-WT CRC cells compared to their parental cells (Figure 6B). Furthermore, we transfected plasmids harboring KRAS^G12D^ into Caco-2 and HT29 cells to create *KRAS*-mutant cells. Interestingly, SLC25A21 increased the CTX sensitivity of KRAS^G12D^-expressing Caco-2 and HT29 cells (Figure 6C), indicating that the CTX sensitivity mediated by SLC25A21 requires *KRAS* mutation. To determine whether SLC25A21 has an antiproliferative effect in combination with CTX, a colony formation assay was performed. Consistently, SLC25A21 overexpression in combination with CTX inhibited the colony formation of *KRAS*-mutant CRC cells (Figure 6, D and E).

Subsequently, we knocked down *SUCLG2* expression with siRNA in CRC cells with SLC25A21 depletion to repress GTP production. The CCK8 and colony formation assay results showed that *SUCLG2* knockdown completely overcame the CTX resistance of *KRAS*-mutant CRC cells with SLC25A21 depletion but not *KRAS*-WT HT29 cells (Figure 6, F and G). In summary, these data suggest that decreasing GTP production by overexpressing SLC25A21 to reduce the available raw material for the conversion of α-KG to succinate and/or inhibiting SCS activity inhibits KRAS activity, which may be a potential treatment strategy for CRC patients with *KRAS* mutations.

### SLC25A21 downregulation results from the inhibition of α-KG–dependent DNA demethylases induced by arrest of α-KG efflux.

**Figure 7:**
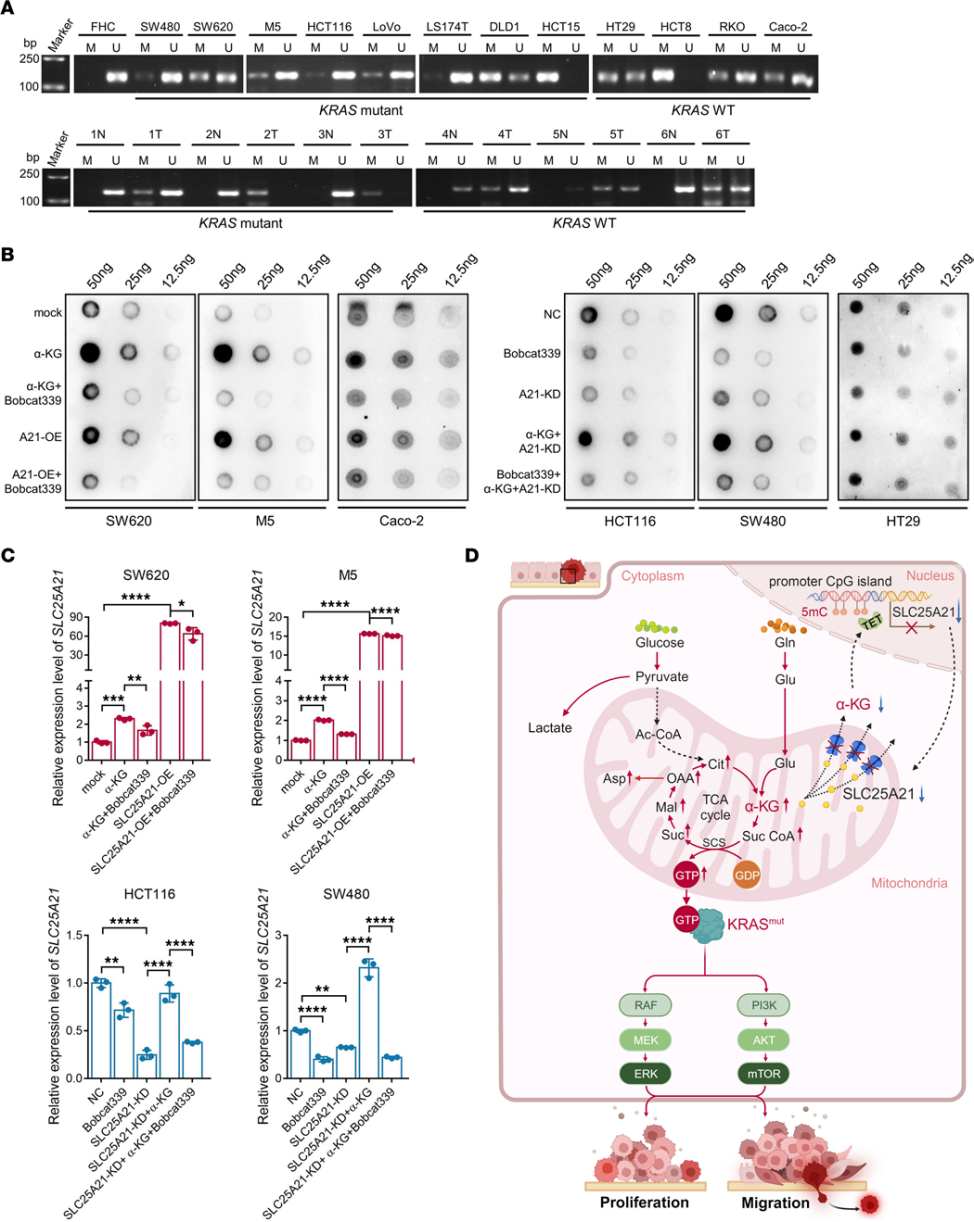
*SLC25A21* downregulation results from the inhibition of α-KG–dependent DNA demethylases induced by arrest of α-KG efflux. (**A**) Analyses of the methylation status of the *SLC25A21* gene in FHC and CRC cell lines, and CRC and paired normal tissue samples ascertained by methylation-specific PCR. Top: FHC cells and *KRAS*-mutant and *KRAS*-WT CRC cells (*n* = 13). Bottom: CRC with *KRAS* mutations or WT *KRAS* and paired normal tissues (*n* = 6). U, unmethylated; M, methylated; N, normal; T, tumor. (**B**) DNA dot blots assessing the 5-hmC levels of *KRAS*-mutant and *KRAS*-WT CRC with or without SLC25A21 overexpression or SLC25A21 knockdown in the absence or presence of α-KG (2 mM) or Bobcat339 (80 μM) for 48 hours. The blot images are representative of 2 independent experiments. (**C**) Relative expression of *SLC25A21* in *KRAS*-mutant CRC cells with or without SLC25A21 overexpression or SLC25A21 knockdown in the absence or presence of α-KG (2 mM) or Bobcat339 (80 μM) for 48 hours (*n* = 3 biologically independent experiments). Data are represented as mean ± SD. Statistical significance was calculated by 1-way ANOVA with Tukey’s post hoc test. *P < 0.05; **P < 0.01; ***P < 0.001; ****P < 0.0001. (**D**) Schematic illustration showing the mechanism of action of SLC25A21 in *KRAS*-mutant CRC. The illustration was created with BioRender.

To explore the underlying mechanisms that contribute to *SLC25A21* downregulation in CRC, we performed a computational screen. Analysis of ENCODE data identified a 647-bp CpG island and higher methylation within the *SLC25A21* promoter region. Methylation-specific PCR was conducted to assay CpG methylation in the *SLC25A21* promoter. As shown in Figure 7A, an unmethylated band was found in FHC cells, whereas a methylated band was observed in both *KRAS*-mutant and *KRAS*-WT CRC cells. We next expanded the methylation analysis to CRC tissue samples and found that the methylation levels of *SLC25A21* were significantly elevated in *KRAS*-mutant CRC tissues compared with normal tissues. A similar result was observed in *KRAS*-WT CRC tissues (Figure 7A). In addition, treatment with 5-aza-2′-deoxycytidine (5-Aza-dC), a DNA demethylation agent, or the methyl group donor *S*-adenosylmethionine (SAM) led to an increase and decrease in *SLC25A21* mRNA in CRC cells, respectively (Supplemental Figure 9). These data indicate that DNA hypermethylation of the *SLC25A21* promoter is critical for the observed *SLC25A21* downregulation in CRC.

α-KG is a pleiotropic compound that serves as a metabolite and as a cofactor for numerous enzymes. The ten-eleven translocation (TET) family is a group of DNA demethylases that are dioxygenases requiring α-KG as a cofactor (28). We hypothesized that *SLC25A21* downregulation inhibits TET demethylase activity by repressing α-KG efflux in CRC. Dot blot assays confirmed that SLC25A21 overexpression increased the abundance of 5-hmC marks to equal that observed with α-KG addition, and this effect was restored by treatment with Bobcat33, a TET1/2 inhibitor (29), in *KRAS*-mutant CRC cells (Figure 7B). In contrast, SLC25A21-depletion-mediated TET inhibition decreased the abundance of 5-hmC marks compared with those in the control, and the effect was equal to that observed with Bobcat339 treatment. The inhibition of 5-hmC deposition by SLC25A21 depletion was reversed by α-KG addition (Figure 7B). In *KRAS*-WT CRC cells, both α-KG addition and Bobcat339 treatment changed the abundance of 5-hmC marks, whereas SLC25A21 knockdown only exerted a slight effect (Figure 7B), which was consistent with the extent through which SLC25A21 downregulation mediated mitochondrial α-KG efflux (Figure 4A). Moreover, we examined the changes in *SLC25A21* expression. Similar to the effect of SLC25A21 overexpression, α-KG supplementation to the *KRAS*-mutant CRC cell culture medium induced the transcription of *SLC25A21*, and this effect was abrogated by Bobcat339 treatment (Figure 7C). In contrast, the *SLC25A21* transcript levels were reduced by inhibition of TET activity, and the effect was similar to that observed with SLC25A21 knockdown that was reversed by α-KG supplementation (Figure 7C). Collectively, these results indicate a positive feedback mechanism involving *SLC25A21* expression and α-KG–dependent TETs in CRC cells.

## Discussion

Cancer cells undergo metabolic adaptations by altering metabolic activities and pathways to meet the requirements for rapid growth and dissemination (30). Emerging evidence shows that *KRAS*-mutated CRC is dependent on Gln for survival and progression (12, 17). However, how Gln metabolism reprogramming promotes and coordinates *KRAS*-mutant CRC growth and progression is largely unknown. In the present study, we identified what we believe are novel functions of SLC25A21 in regulating Gln metabolism in *KRAS*-mutant CRC. We present data showing that SLC25A21 expression was downregulated in *KRAS*-mutant CRC tissues and cell lines and that *SLC25A21* downregulation was correlated with poor survival of patients with *KRAS*-mutant CRC. SLC25A21 downregulation selectively accelerated the growth, invasion, migration, and metastasis of *KRAS*-mutant CRC cells in vitro and in vivo. Furthermore, we discovered that SLC25A21 downregulation inhibited Gln-derived α-KG efflux in mitochondria to potentiate glutaminolysis. This arrested α-KG efflux in response to SLC25A21 downregulation enhanced downstream oxidative decarboxylation reactions and GTP production and thereby induced persistent KRAS activation in *KRAS*-mutant CRC. In addition, the restoration of SLC25A21 expression abrogated mutant-*KRAS*-mediated CTX resistance in CRC. Moreover, we discovered that the arrest of α-KG efflux mediated by SLC25A21 depletion inhibited α-KG–dependent DNA demethylase activity, which further decreased *SLC25A21* expression (Figure 7D).

The SLC superfamily carries a wide variety of substances across cellular membranes (31). Recent evidence has shown that the aberrant expression of SLCs can promote tumor growth and dissemination by acquiring the necessary metabolic substrates and modulating the way nutrients are used (12, 18, 32). For example, SLC25A22 promotes *KRAS*-mutant CRC cell proliferation and survival by increasing Glu influx into mitochondria and thereby promoting Gln metabolism (12). SLC25A8-mediated aspartate transport is required to support PDAC growth (19). We comprehensively analyzed the changes in the expression of SLC transporters associated with glutaminolysis metabolic flux using TCGA data and found that the aberrant expression of SLCs is a common feature of *KRAS*-mutant CRC, indicating that metabolite transporters may be a novel biomarker as well as a potential therapeutic target in *KRAS*-mutant CRC. Among such transporters, we focused on the SLC25A21 transporter, which exhibited downregulated expression in *KRAS*-mutant CRC. Gene expression analysis showed that *SLC25A21* was downregulated in 2 of 7 nasopharyngeal carcinoma cell lines (21). A recent study demonstrated that low expression of *SLC25A21* predicted unfavorable prognosis in patients with acute myeloid leukemia (33). However, the precise biological functions of SLC25A21 and the mechanisms underlying its dysregulation in cancer remain largely unknown. In the present study, we comprehensively confirmed the dramatic inhibitory effects of SLC25A21 on *KRAS*-mutant CRC through a series of in vitro and in vivo functional experiments. Interestingly, we found that SLC25A21 depletion inhibited cell growth in a *KRAS*-mutation-dependent manner. Clinical prognostic data from TCGA showed that *SLC25A21* downregulation was correlated with poor survival of CRC patients harboring *KRAS* mutation. Together, these results indicate that SLC25A21 downregulation selectively affects the behavior of *KRAS*-mutant CRC cells.

Gln is the most abundant free amino acid in the human body (34). There is increasing evidence that energy production, redox homeostasis, and macromolecular synthesis rely on Gln consumption in most cancer cells (35, 36). Gln is thought to play a crucial role in cancer cells via glutaminolysis mediated by glutaminases, which convert Gln to Glu; Glu can be further deaminated by Glu dehydrogenase or transaminases to generate α-KG. α-KG enters the TCA cycle and undergoes anaplerosis, thereby replenishing metabolic intermediates for energy production and biosynthesis. In the present study, we found that SLC25A21 depletion significantly inhibited mitochondrial α-KG efflux in *KRAS*-mutant CRC cells. Using [U-^13^C_5_]Gln as a tracer to monitor metabolic fluxes in KRAS-mutant CRC cells, we found that SLC25A21 depletion resulted in a significant increase in Gln-derived C4 metabolites, indicating that Gln metabolism is needed to fuel the TCA cycle. A previous study reported that the anaplerotic TCA cycle is an indispensable role of Gln in *KRAS*-mutant cancer (9, 12, 18, 19), which is consistent with our work presented herein. Moreover, we showed that the levels of (m+3) and (m+4) aspartate, as well as those of (m+5) and (m+4) citrate, were significantly increased in SLC25A21-downregulated CRC cells. Given the significant roles of aspartate in amino acid and nucleotide synthesis, and of citrate in de novo lipogenesis, our results support the hypothesis that SLC25A21 downregulation accelerates Gln-derived biosynthesis in *KRAS*-mutant CRC cells. α-KG can act as a true antioxidant because it directly reacts with H_2_O_2_ in mitochondria to form succinate (37). In addition, both malate and oxaloacetate can be catalyzed to produce NADPH, a cofactor for antioxidant enzymes (12, 38). Therefore, our results reveal that SLC25A21 depletion could balance cellular redox homeostasis by promoting the conversion of α-KG to succinate and increasing NADPH production. Moreover, our results reveal that the rescue effects of α-KG supplementation on Gln deprivation depend on SLC25A21 depletion in *KRAS*-mutant CRC, implying that SLC25A21 downregulation is needed for Gln to fuel *KRAS*-mutant CRC cell proliferation. Notably, in *KRAS*-WT CRC cells, SLC25A21 depletion also inhibited mitochondrial α-KG efflux to some extent, but the inhibitory effect was less substantial than that observed in *KRAS*-mutant cells, resulting in no significant downstream effects on Gln-derived α-KG to replenish the TCA cycle and biological function. One explanation could be that *KRAS* mutation can increase the utilization of Gln (9, 39). Collectively, these results reveal the crucial role of SLC25A21-defect-mediated glutamine anaplerosis in *KRAS*-mutant CRC.

Because SLC25A21 is a counter-exchanger and responsible for the transport of 2-OA and α-KG across the IMM, the SLC25A21-depletion-mediated arrest of mitochondrial α-KG would result in the accumulation of 2-OA in the cytosol. Boczonadi et al. simulated the effects of SLC25A21 deficiency using a computer model to investigate the consequences of impaired SLC25A21 carriers, and found increases in pipecolic acid (PA) and quinolinic acid (QA), which are spontaneously formed from semialdehydes (an upstream intermediate of 2-OA in tryptophan degradation) (40). However, accumulation of other intermediates, such as 2-OA, was not observed. In addition, these researchers found increased 2-OA, PA, and QA levels in urine samples derived from SLC25A21-deficient patients through MS-based analysis. These sources indicated that the cytosolic accumulation of 2-OA mediated by SLC25A21 deficiency can be metabolized through alternative pathways or be excreted from the cytosol into bodily fluids, such as urine, although the efflux mechanism remains unknown. Some studies have revealed that the addition of lysine or tryptophan increases the malignant properties of cancer cells (41–43). Because 2-OA is an important intermediate in lysine and tryptophan metabolism pathways, further study monitoring 2-OA metabolic fluxes using a tracer will provide a more comprehensive elucidation of the function of SLC25A21 depletion in *KRAS*-mutant CRC cells.

Despite intensive efforts to identify new effective targets for *KRAS*-mutant CRC, targeting KRAS mutations remains a significant challenge. The different *KRAS* mutations have distinct oncogenic properties and are differentially responsive to targeted therapies (5). The recent development of KRAS^G12C^-specific inhibitors has yielded renewed hope (44). However, the KRAS p.G12C mutation only occurs in 1% to 3% of CRC (45), and other more common KRAS mutations in CRC remain “undruggable.” The high affinity of KRAS for its substrate GTP and the high concentration of GTP in the cells make it exceedingly unlikely that a successful competitive KRAS inhibitor can be developed (46). Therefore, a shift in strategy away from KRAS inhibitors to approaches that aim to enhance the sensitivity to anti-EGFR antibodies in *KRAS*-mutant CRC is warranted. Our results that SLC25A21 downregulation increased GTP production and KRAS activity and subsequently activated the PI3K/AKT and RAF/ERK signaling pathways encouraged us to explore the effect of SLC25A21 dysregulation on the efficacy of anti-EGFR antibody treatment in *KRAS*-mutant CRC. Importantly, we demonstrated the therapeutic value of SLC25A21 because its overexpression sensitized *KRAS*-mutant CRC cells to CTX in vitro by decreasing the level of mitochondrial α-KG, a raw material for GTP production. Similar to the effects of SLC25A21 overexpression, the inhibition of SCS activity induced by *SUCLG2* knockdown overcame the resistance of *KRAS*-mutant CRC cells to CTX. Our results expanded the understanding of glutaminolysis in *KRAS*-driven cancers by providing GTP for activating KRAS, which suggests that selectively starving cancer cells by reducing GTP, for example, by overexpressing SLC25A21 or inhibiting SCS, is a potential treatment strategy for CRC patients harboring *KRAS* mutation. However, because GTP binds KRAS protein with picomolar affinity and given the physiological roles of GTP in normal cells, the metabolic pathways by which GTP production is limited to inhibit cancer progression need to be better understood for the design of effective drugs. Moreover, our study suggested that SLC25A21 overexpression has potential as a CTX response marker for *KRAS*-mutant CRC. Therefore, the development of possible approaches for increasing synergistic growth inhibition with anti-EGFR therapy for cancer cells may provide tractable therapeutic opportunities for future drug development initiatives.

Despite previous studies reporting that KRAS activation induces the expression of genes (47–49), our results showed that *SLC25A21* expression was heterogeneous in both *KRAS*-mutant and *KRAS*-WT CRC. No increase in SLC25A21 expression was observed upon acquisition of *KRAS* mutation, which indicates that SLC25A21 expression is not regulated in a *KRAS*-dependent manner. Cancer metabolism is closely associated with epigenetic regulation because metabolites are needed as cofactors and substrates for epigenetic modifying enzymes (28). Some studies have reported that TCA cycle intermediates, such as α-KG, succinate, and fumarate, can directly contribute to global genomic epigenetic regulation (22, 50). TETs are α-KG–dependent dioxygenases that may facilitate DNA demethylation (28, 51). The abundance of α-KG can directly affect TET activity, whereas succinate and fumarate are structurally similar to α-KG and can therefore competitively inhibit TET activity (50). Our results showed that DNA hypermethylation of the *SLC25A21* promoter is critical for the observed *SLC25A21* downregulation in CRC. In addition, our findings showed that the inhibitory effect on TET activity mediated by SLC25A21 depletion was abolished by α-KG supplementation, suggesting that positive feedback exists between *SLC25A21* expression and TET activity mediated by SLC25A21-induced α-KG efflux in CRC cells. Our results strengthen the link between metabolism and epigenetic dysregulation and elucidate light on newly discovered connections between substrate consumption and carcinogenesis.

In summary, we demonstrated that SLC25A21, a mitochondrial transporter, plays important roles in *KRAS*-mutant CRC by affecting metabolite transport, namely, Gln-derived α-KG efflux, and subsequent Gln metabolism and GTP production. Moreover, increasing SLC25A21 expression can attenuate *KRAS*-mutation-mediated resistance to CTX in *KRAS*-mutant CRC. In summary, our data illustrate the importance of SLC25A21 dysregulation in rewiring tumor metabolism in *KRAS*-mutant CRC and inform therapeutic strategies for targeting CRC with *KRAS* mutation.

## Methods

### Tissue specimens and cell culture.

Tissue samples were obtained from patients with a diagnosis of primary CRC who then underwent elective surgery at Nanfang Hospital, Southern Medical University. Fresh CRC and paired nontumor mucosal tissues were freshly frozen and stored in liquid nitrogen. A cohort of 141 archived CRC tissue samples with *KRAS* mutations or WT *KRAS* were collected and used to analyze SLC25A21 expression by IHC.

The human CRC cell lines HCT116, HT29, LS174T, SW480, HCT15, RKO, Caco-2, LoVo, M5, SW620, HCT8, and DLD1, and the immortalized colonic mucosal epithelial cell line FHC, were obtained from the American Type Culture Collection (ATCC). The M5 cell line, a subclone from SW480 cells with enhanced liver metastasis, was isolated in our laboratory (52). All CRC cell lines were cultured in RPMI 1640 medium (Invitrogen) containing 10% fetal bovine serum (FBS) (Invitrogen) at 37°C under 5% CO_2_. FHC cells were cultured in DMEM/F12 (Invitrogen) with 10% FBS, 25 mM/L HEPES, 100 mg/L hydrocortisone, 5 mg/L insulin, 10 mg/L cholera toxin, 5 mg/L transferrin, and 20 mg/L human recombinant EGF. PDAC cell lines were provided by Zhi-Yong Liang (Peking Union Medical College Hospital, Beijing, China).

### Construction of cell lines with stable overexpression or downregulation of SLC25A21.

The full-length human *SLC25A21* DNA fragment was amplified and cloned into the LV5 (EF-1aF/GFP/Puro) lentiviral vector (GenePharma). *SLC25A21* short hairpin RNA (shRNA) and negative control (NC) shRNA were purchased from GeneChem and inserted into the GV248 lentiviral vector (GeneChem). Virus particles were harvested 48 hours after corresponding vector transfection into cells using Lipofectamine 3000 reagent (Thermo Fisher Scientific). Cells were infected with the appropriate recombinant lentivirus transducing units and then selected with puromycin to obtain CRC cells with stable overexpression or downregulation of SLC25A21. The empty lentiviral vector LV5 or the GV248 vector with NC shRNA was used as the corresponding control. The primers and target sequences for shRNAs are shown in Supplemental Table 1.

### Generation of CRC cells with KRAS mutation.

The pUC57 plasmid (Addgene, 54338) expressing KRAS^G12D^ was obtained from Amoy Diagnostics and then transfected into HT29 and Caco-2 cells, which are 2 *KRAS*-WT CRC cell lines, using Lipofectamine 3000 to generate *KRAS*-mutated HT29 and KRAS-mutated Caco-2 cells.

### Functional assays in vitro and in vivo.

Cell proliferation, colony formation, flow cytometry, wound-healing, and invasion assays in vitro and tumorigenic and metastasis assays were performed according to standard protocols as described previously (53, 54). Further details are provided in the Supplemental Methods.

### Human primary CRC organoid construction, culture, and treatment.

Fresh surgically resected CRC tissues were washed with Dulbecco’s PBS supplemented with penicillin/streptomycin and 10 μM Y-27632 (MedChemExpress, HY-10583), cut into 1-mm^3^ pieces, and then enzymatically dissociated using a tissue pretreatment kit (Accurate Int., K301). The collected cells were washed and resuspended in advanced DMEM/F12 medium (Invitrogen) and embedded in growth factor–reduced Matrigel (Corning, catalog 356231) at a ratio of 1:1.5. The dome was incubated at 37°C for 10 minutes. After solidification, the organoid culture medium, which consisted of advanced DMEM/F12 medium with 20 mM HEPES, 2 mM GlutaMax, B27 supplement, 10 mM nicotinamide, 50 ng/mL EGF, 10 nM gastrin, 100 ng/mL Wnt3a, 100 ng/mL R-spondin-1, 100 ng/mL Noggin, 500 nM A83-01, 1.25 mM *N*-acetyl cysteine, and penicillin/streptomycin, was added and replenished every 2–3 days. Organoids were digested into a single-cell suspension, infected with LV5-*SLC25A21* or LV5 lentivirus for 4 hours at 37°C, and reconstituted in Matrigel.

### KRAS activity assay.

RAS-GTP levels in cell lysates were assessed using a K-Ras activation assay kit (Abcam, ab211159). Briefly, Ras-GTP from cell lysates was isolated and pulled down selectively using Raf1 RBD agarose beads (Abcam, ab211159). Subsequently, the precipitated GTP-Ras was detected by Western blotting using an anti–K-Ras polyclonal antibody (Abcam, ab180772; 1:1000 dilution).

Protocols for other procedures are provided in the Supplemental Methods.

### Data availability.

The RNA-sequencing data, mutation data, and clinical information of CRC samples were obtained from TCGA (https://www.cancer.gov/tcga) via TCGAbiolinks R/Bioconductor package (version 2.2.0) (55). All data supporting the findings of this study and its supplemental results are available in the Supporting Data Values file.

### Statistics.

All statistical analyses were performed using SPSS Statistics version 22 (IBM). The statistical analyses included 2-tailed Student’s *t* test, Fisher’s exact test, and 1-way and 2-way ANOVA. Survival curves were plotted by the Kaplan-Meier method and compared by the log-rank test. A *P* value of less than 0.05 was defined as statistically significant.

### Study approval.

The use of clinical tissue specimens for this study was approved by the ethics committee of Nanfang Hospital, Southern Medical University (Guangzhou, China; no. NFEC-2021-108). All of the patients signed an informed consent form before their clinical materials were used for research purposes. Moreover, the animal experiments were performed in strict accordance with the recommendations in the NIH *Guide for the Care and Use of Laboratory Animals* (National Academies Press, 2011). The protocol was approved by the Animal Ethics Committee of Nanfang Hospital, Southern Medical University (no. NFYY-2019-1118).

## Author contributions

SSH and SW conceptualized the study. SSH, YH, and TYT designed the methodology. SSH, YH, TYT, HC, JWG, LZ, LW, and YQW contributed to the experimental procedures. SSH, YH, TYT, and SW conducted the analysis. SW, MHY, and YQD acquired funding, provided resources, and supervised all the work. All authors contributed to the manuscript review and editing process.

## Supplementary Material

Supplemental data

Supporting data values

## Version 1 11/08/2023

Electronic publication
