# Supplementary material for: SLC25A21 downregulation promotes KRAS-mutant colorectal cancer progression by increasing glutamine anaplerosis
Source: JCI Insight. 2023 Nov 8;8(21):e167874. doi: 10.1172/jci.insight.167874 (PMC10721270; doi:10.1172/jci.insight.167874)
Supplement: Supplemental data [file jciinsight-8-167874-s030.pdf]

# Supplementary Figures

A

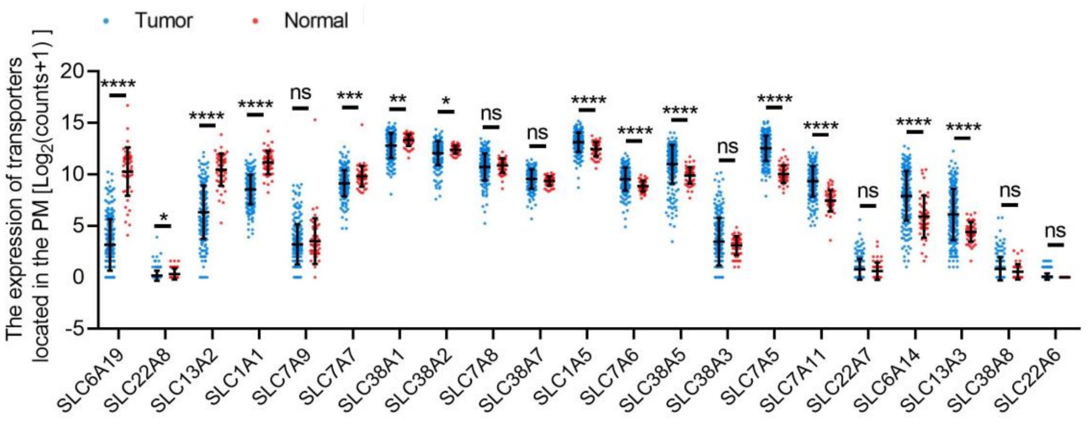


B

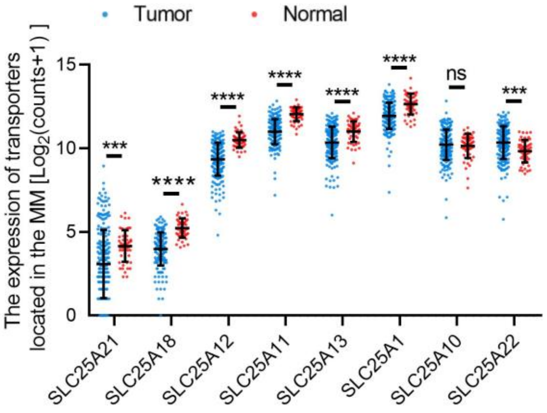


C

| Gene | Location | Substrates | Fold Change (T/N) | $Log_2FC$ |
|---|---|---|---|---|
| SLC25A22 | MM | glutamate | 1.051509279 | 0.07246 |
| SLC25A21 | MM | 2-oxoadipate/2-ketoglutarate | 0.741184769 | -0.43209 |
| SLC25A18 | MM | glutamate | 0.761455877 | -0.39317 |
| SLC25A12 | MM | aspartate/glutamate | 0.890198803 | -0.1678 |
| SLC25A11 | MM | 2-ketoglutarate/malate | 0.913057179 | -0.13122 |
| SLC25A13 | MM | aspartate/glutamate | 0.940563971 | -0.0884 |
| SLC25A1 | MM | citrate/malate | 0.944597555 | -0.08223 |
| SLC25A10 | MM | malate, succinate | 1.006913871 | 0.00994 |

Figure S1. Transcript abundance of SLC transporters of gluatminolysis-accociated metabolites in human *KRAS*-mutant CRC samples using publicly available databases from TCGA. (A and B) The mRNA expression of 29 SLC transporters locates to the plasma (A) and mitochondrial (B) membrane in normal and cancer samples (tumor, *n* = 231; normal, *n* = 51). (C) The information of mitochondrial SLC transporters. PM, plasma membrane; MM, mitochondrial membrane; ns, nonsignificant. Data are represented as mean ± SD. Statistical significance was calculated by unpaired two-sided *t* test (A and B). *$P$<0.05, **$P$<0.01, ***$P$<0.001, and ****$P$<0.0001.

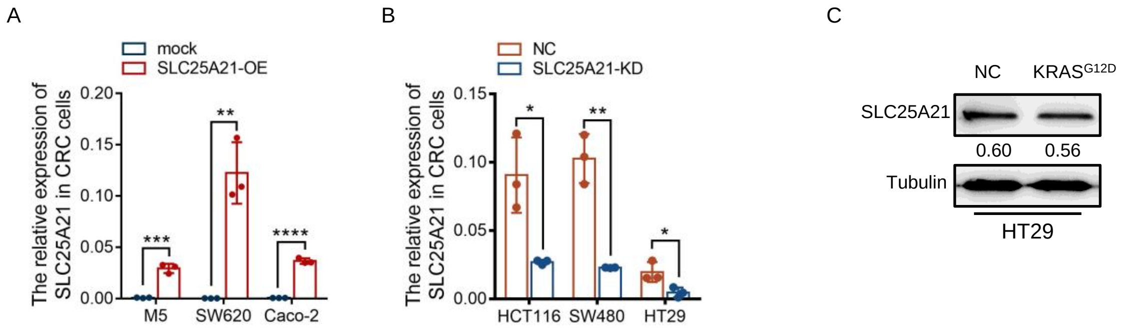


Figure S2. The expression levels of SLC25A21 in CRC cells. (A and B) Relative transcript levels of *SLC25A21* in CRC cells with or without SLC25A21 overexpression (A) or SLC25A21 knockdown (B) by real-time RT-PCR (*n* = 3 biologically independent experiments). (C) Immunoblot analysis of SLC25A21 protein in $KRAS^{G12D}$-introduction HT29 cells. Data are presented as the mean ± SD. Statistical significance was calculated unpaired 2-sided *t* test (A and B). *$P$<0.05, **$P$<0.01, ***$P$<0.001, and ****$P$<0.0001.

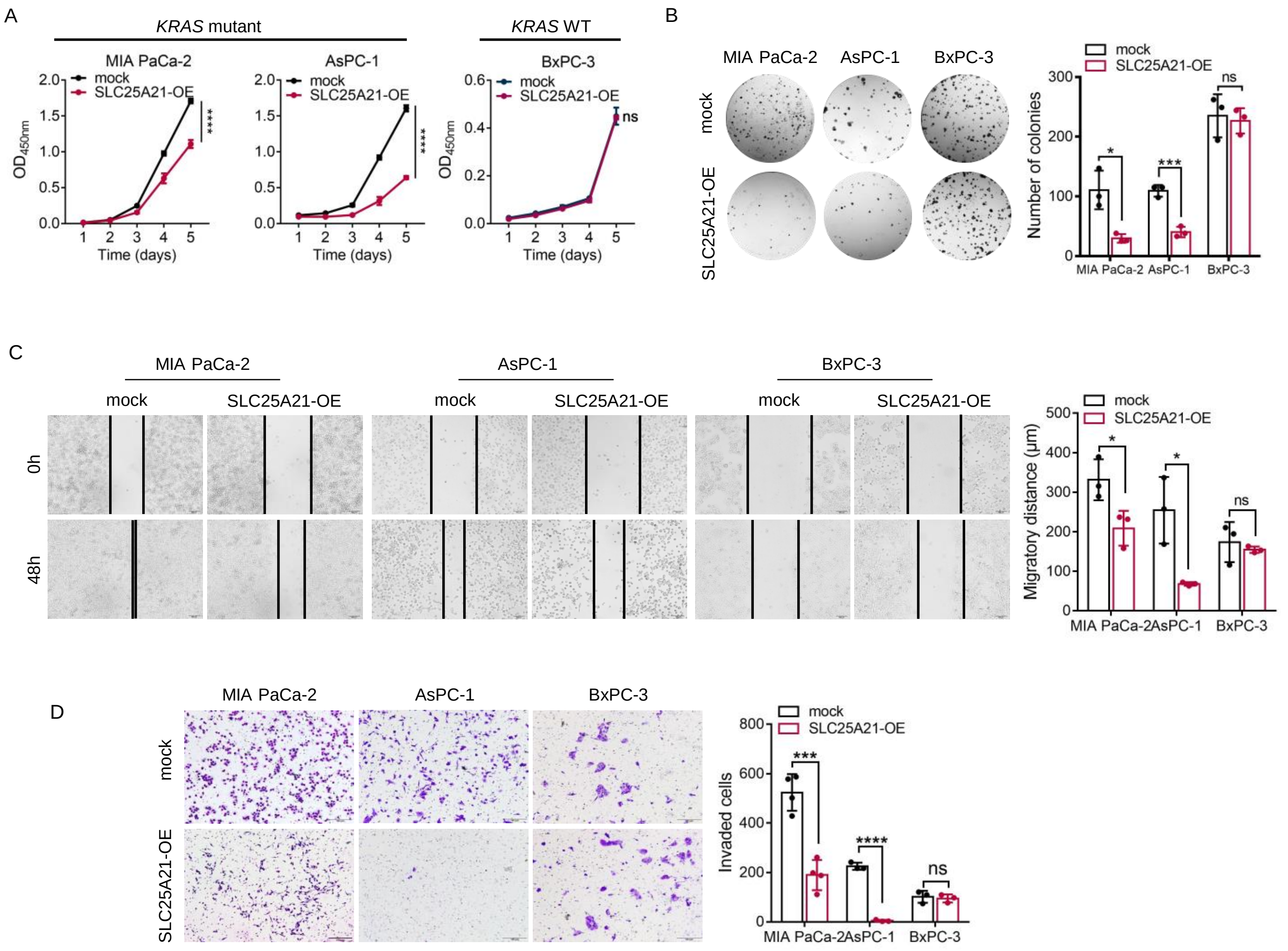


Figure S3. SLC25A21 overexpression selectively inhibits cell growth, migration and invasion of *KRAS*-mutant PDAC in a KRAS mutation-dependent manner. (A) Cell proliferation ability of *KRAS*-mutant and *KRAS*-WT PDAC cells with or without SLC25A21 overexpression (*n* = 3 biologically independent experiments). (B) Representative images (left) and quantification (right) showing the colony-forming capacity of *KRAS*-mutant and *KRAS*-WT PDAC cells with or without SLC25A21 overexpression (*n* = 3 biologically independent experiments). (C and D) Representative images and quantification showing the migration (C) and invasion capacity (D) of *KRAS*-mutant and *KRAS*-WT PDAC cells with or without SLC25A21 overexpression (*n* = 3-4 biologically independent experiments). Left, representative images; Scale bars, 100μm. right, quantification analyses. *KRAS* WT, *KRAS* wild-type; SLC25A21-OE, SLC25A21 overexpression; ns, nonsignificant. Data are presented as the mean ± SD. Statistical significance was calculated by two-way ANOVA followed by correction for multiple comparisons (A), unpaired two-sided *t* test (B-C). *$P$<0.05, ***$P$<0.001, and ****$P$<0.0001.

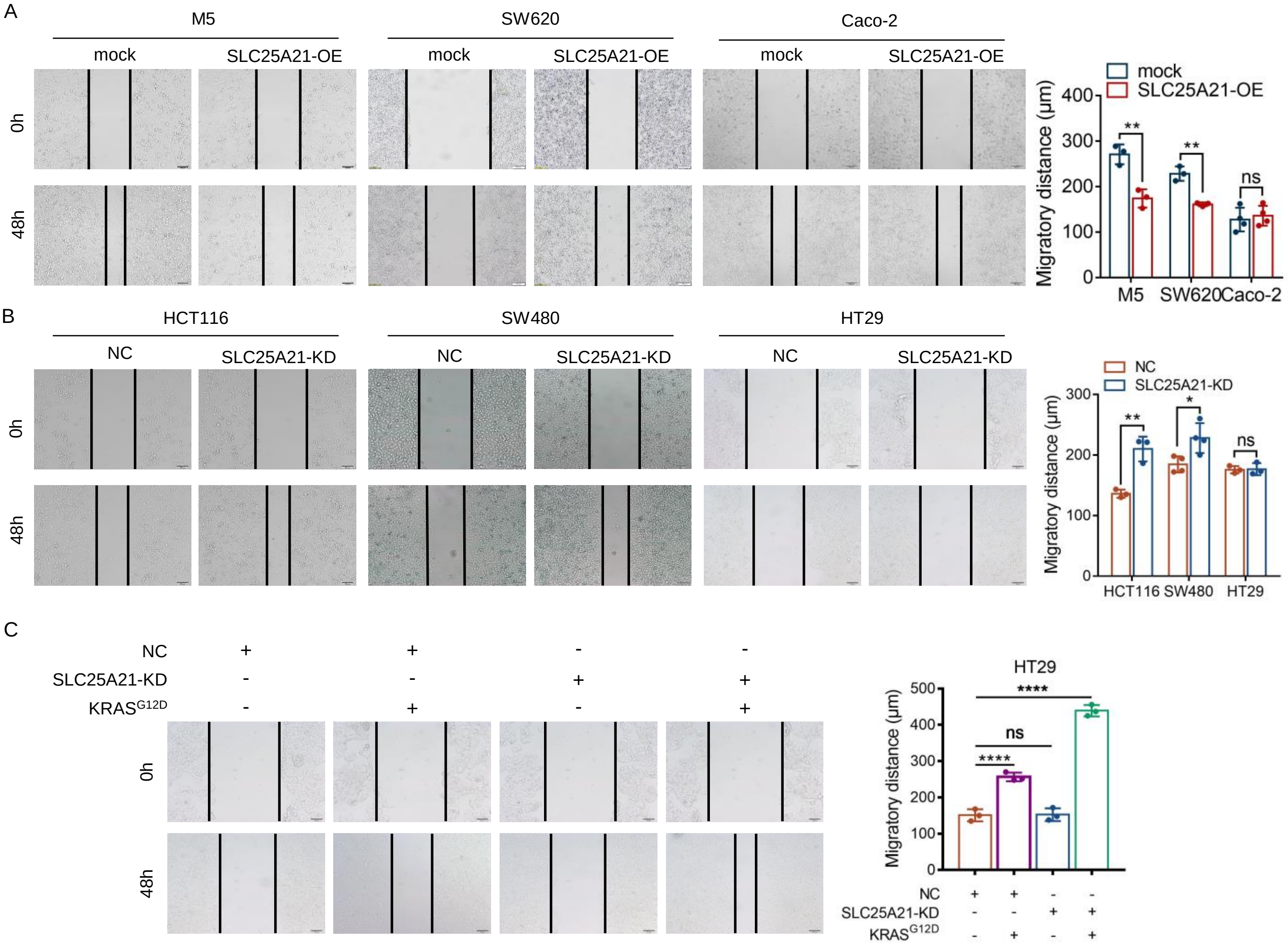


Figure S4. SLC25A21 inhibits the migration of *KRAS*-mutant CRC cells in a *KRAS* mutation-dependent manner in vitro. (A and B) Representative images (left) and quantification (right) showing the migration capacity of *KRAS*-mutant and *KRAS*-WT CRC cells with or without SLC25A21 overexpression (A) or SLC25A21 knockdown (B, $n$ = 3-4 biologically independent experiments). (C) Representative images (left) and quantification (right) showing the invasiveness of HT29 cells with or without expression of mutated $KRAS^{G12D}$ or SLC25A21 knockdown ($n$ = 3 biologically independent experiments). Data are presented as the mean ± SD. Statistical significance was calculated by unpaired two-sided $t$ test (A and B) and one-way ANOVA with Dunnett's post hoc test (C). *$P$<0.05, **$P$<0.01, ***$P$<0.001, and ****$P$<0.0001.

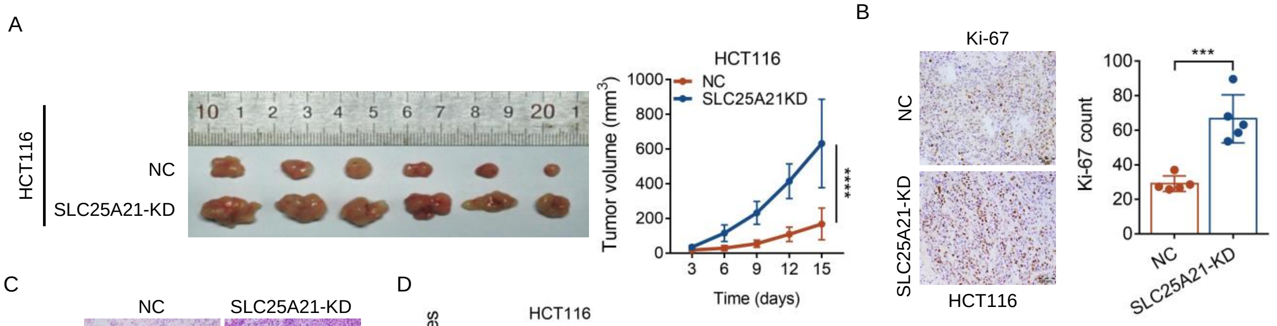


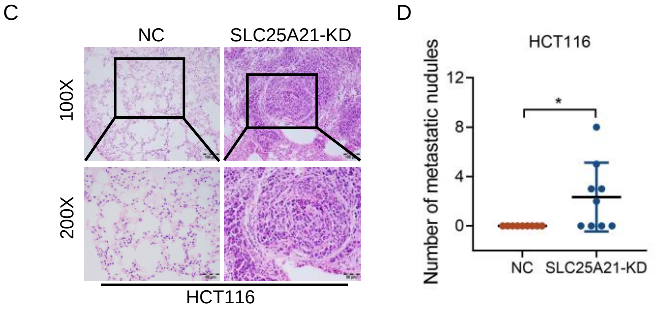


E

| Group | With lung metastasis | *P* |
|---|---|---|
| NC | 0/9 | 0.0294 |
| SLC25A21-KD | 5/9 | |

Figure S5. SLC25A21 depletion promoted the tumorigenicity and metastasis of *KRAS*-mutant CRC cells in vivo. (A) Bright-field images of tumors (left) and growth curves of tumor volume (right) from nude mice ectopically transplanted with HCT116 cells with or without SLC25A21 depletion ($n$ = 6 per group). The graphs show data from the tumor xenografts at 15 days after cells were ectopically and subcutaneously implanted. (B) Representative IHC staining (left) and quantification (right) showing Ki67 index of tumor xenografts from CRC cells. Scale bars, 50 μm. (C) Representative H&E staining images showing lung metastases from nude mice 4 weeks after tail vein injection of CRC cells with or without SLC25A21 depletion ($n$ = 9 per group). Scale bars, 100μm or 50 μm. (D) Quantification of pulmonary tumor colonies after tail vein injection of HCT116 cells with SLC25A21 depletion or control cells. (E) Statistical comparisons of lung metastases after tail vein injection of SLC25A21-depletion and control cells ($n$ = 9 per group). Data are presented as the mean ± SD. Statistical significance was calculated by 2-way ANOVA (A), unpaired 2-sided $t$ test (B and D) and Fisher's exact test (E). *$P$<0.05, **$P$<0.01, ***$P$<0.001, and ****$P$<0.0001.

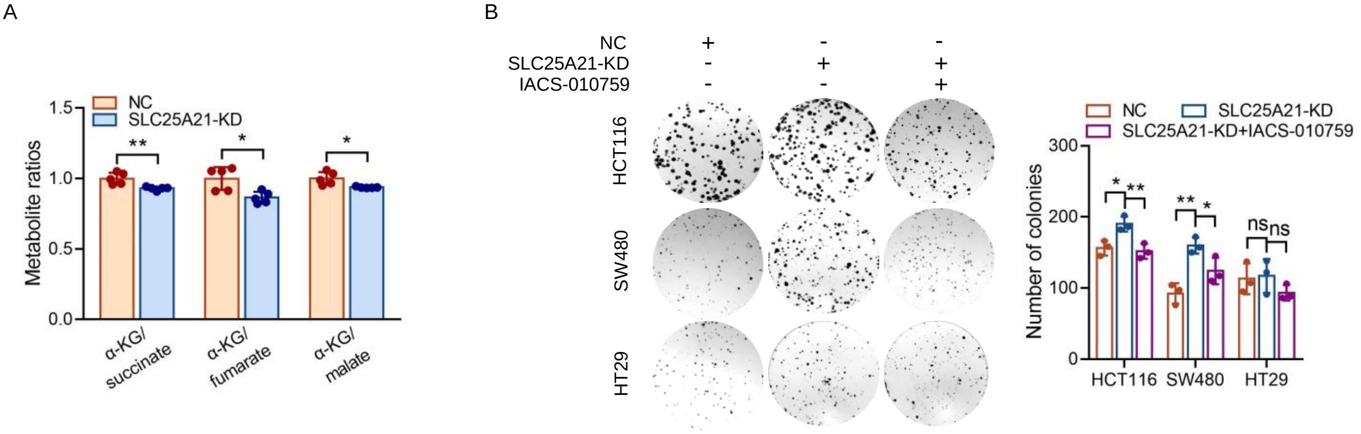


Figure S6. SLC25A21 promotes oxidative phosphorylation in *KRAS*-mutant CRC cells. (A) The ratios of intracellular metabolites of HCT116 cells with or without *SLC25A21* knockdown (*n* = 5 biologically independent samples). (B) Representative images (left) and quantification (right) of the colony-forming capacity of CRC cells with or without *SLC25A21* knockdown in the absence or presence of IACS-010759 (100 nM) for 48 h (*n* = 3 biologically independent experiments). Data are presented as the mean ± SD. Statistical significance was calculated by unpaired two-sided *t* test (A) and one-way ANOVA (C). *$P<0.05$ and **$P<0.01$.

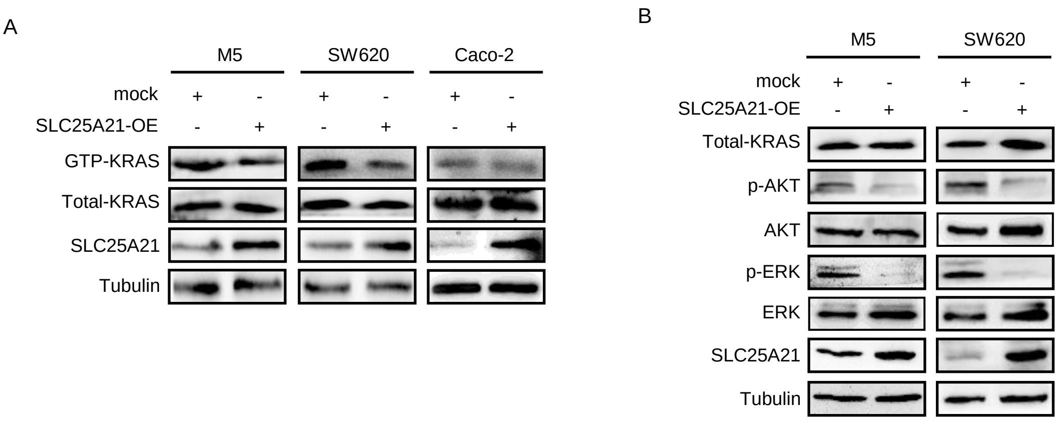


Figure S7. SLC25A21 overexpression inhibits the KRAS activity and AKT/ERK signaling pathways. (A) Immunoblot analysis of KRAS activity of *KRAS*-mutant and *KRAS*-WT CRC cells with or without SLC25A21 overexpression. (B) Immunoblot analysis of the activity of KRAS/ERK/AKT pathways in *KRAS*-mutant CRC cells with or without SLC25A21 overexpression. Blot images shown are representative of two independent experiments.

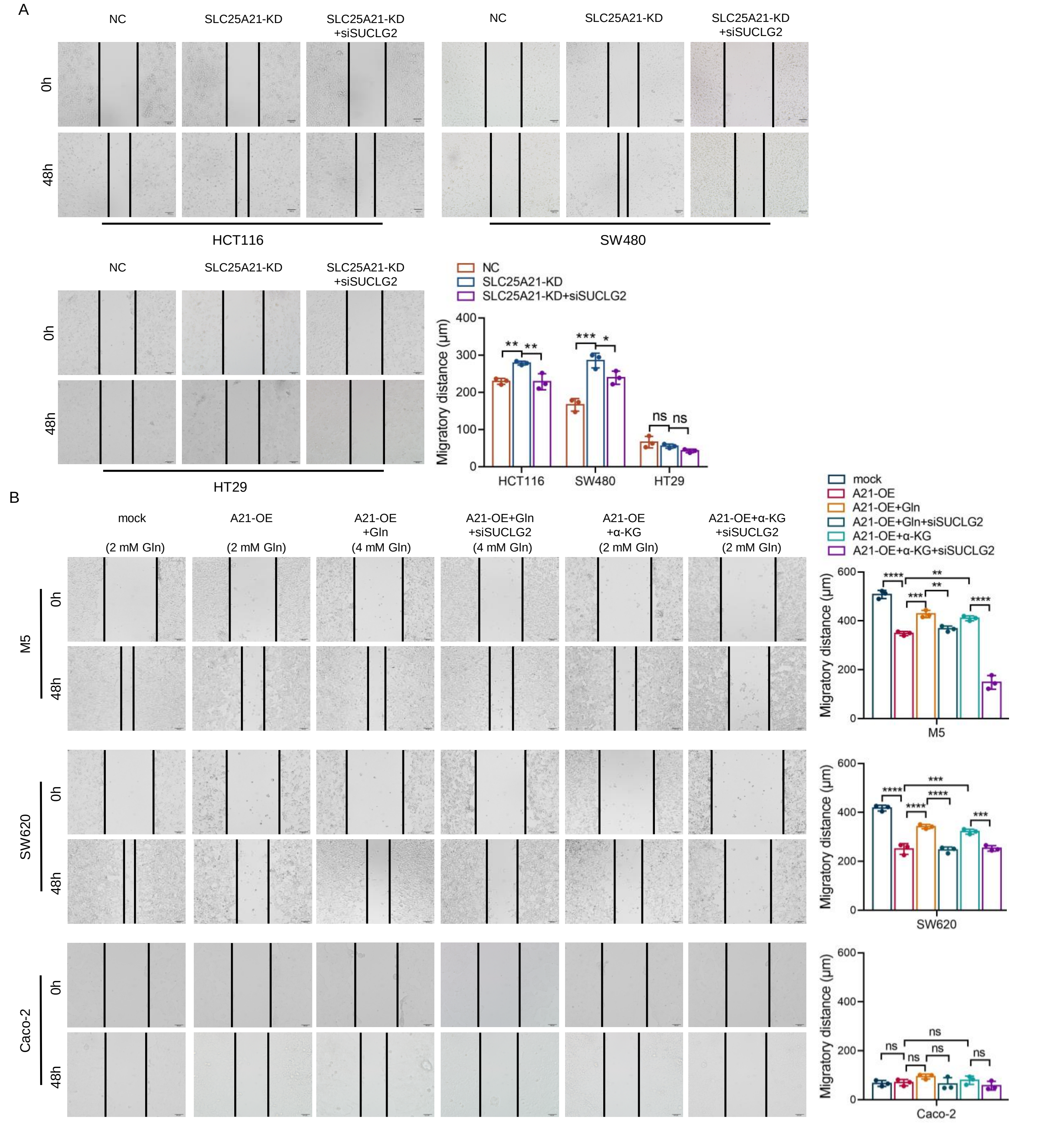


Figure S8. The effects of SLC25A21 depletion on cell migration require SUCLG2 in *KRAS*-mutant CRC. (A) Representative images and quantification showing the migration capacity of *KRAS*-mutant and *KRAS*-WT CRC cells with or without SLC25A21 or SUCLG2 knockdown, treated as shown. Top and bottom left, representative images; bottom right, quantification analyses ($n$ = 3 biologically independent experiments). (B) Representative images (left) and quantification (right) of the migration capacity of CRC cells with or without SLC25A21 overexpression or SUCLG2 downregulation in the absence or presence of α-KG or Gln addition, treated as shown ($n$ = 3 biologically independent experiments). Cells were cultured in normal medium (Gln-containing, 2 mM) or conditioned medium with α-KG addition (2 mM) or Gln addition (total in 4 mM). Data are represented as mean ± SD. Statistical significance was calculated by one-way ANOVA followed by correction for multiple comparisons (A and B). **P*<0.05, ***P*<0.01, ****P*<0.001, and *****P*<0.0001.

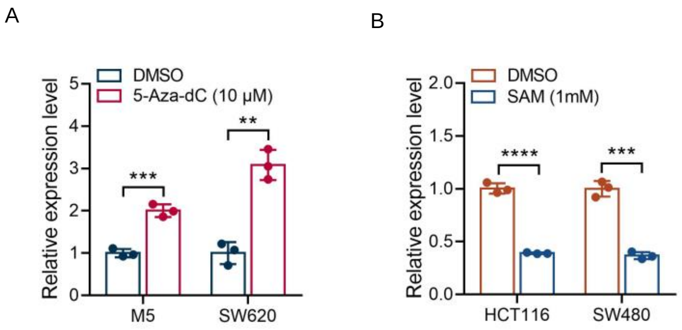


Figure S9. *SLC25A21* expression was regulated by DNA methylation. (A and B) Relative transcript levels of *SLC25A21* mediated by the DNA demethylation agent 5-Aza-dC (A) and the methyl group donor SAM (B) determined by real-time RT-PCR ($n$ = 3 biologically independent experiments). Data are represented as mean ± SD. Statistical significance was calculated by unpaired two-sided $t$ test (A and B) . **$P$<0.01, ***$P$<0.001, and ****$P$<0.0001.

## Supplementary Materials and Methods

### RNA extraction and real-time PCR

Total RNA was extracted from tissue samples and cell lines using TRIzol reagent (catalog 9109, TaKaRa, Dalian, China) according to the manufacturer’s instructions. cDNA was synthetized using the Prime-Script RT Reagent Kit (catalog RR036, TaKaRa). Real-time PCR was performed using SYBR Premix Ex Taq II (catalog RR420, TaKaRa) and the ABI PRISM 7500 Sequence Detection System (Applied Biosystems). The assay was performed in triplicate for each case to allow assessment of the technical variability. The results were normalized to the expression of GAPDH. The primer sequences are listed in Supplementary Table S2.

### Western blotting analysis

Tissues and cells were lysed in prechilled RIPA buffer containing phosphatase inhibitors, protease inhibitors and PMSF. The protein lysates were separated by 10% SDS-PAGE and electrotransferred from the gel to polyvinylidene fluoride (PVDF) membranes (catalog IPVH00010, Millipore, armstadt, Germany). After blocking in 5% BSA solution in 1x PBS-T (0.5% Tween-20) for 1 h, the membranes were incubated with the following primary antibodies: anti-SLC25A21 (catalog DF4172, Affinity Biosciences, OH, USA; 1:1000 dilution), anti-pAKT (catalog AF0016, Affinity Biosciences; 1:1000 dilution), anti-AKT (catalog AF6261, Affinity Biosciences; 1:1000 dilution), anti-pERK (catalog AF1015, Affinity Biosciences; 1:1000 dilution), anti-ERK (catalog AF0155,

Affinity Biosciences; 1:1000 dilution) and anti-tubulin (catalog AF4351, Affinity Biosciences; 1:1000 dilution), as protein-loading controls at 4°C overnight. The blots were incubated with HRP-conjugated secondary antibodies at room temperature for 1 h and visualized with ECL Western blotting Substrate (Thermo Fisher Scientific).

**TCGA analysis**

TCGA data set was used to analyze the levels of SLC25A21 expression in human tumors and the relationship between SLC25A21 expression and the survival time of 231 KRAS-mutant CRC patients.

**Oligonucleotide transfection**

siRNAs targeting SUCLG2 and NC siRNA were synthesized (GenePharma). Oligonucleotide transfection was performed with Lipofectamine 3000 (catalog L3000-015, Thermo Fisher Scientific) following the manufacturer's protocol. The target sequences for siRNAs are shown in Supplementary Table S3.

**Cell proliferation and colony formation assays**

For the cell proliferation assay, cells were seeded into 96-well plates at $1\times10^3$ cells per well. After 24 h, the cell proliferation ability was evaluated using CCK-8 (catalog CK04-3000T, Dojindo Laboratories, Kyushu Island, Japan) according to the manufacturer's protocol.

For the colony formation assay, cells were plated in 6-well plates at $5 \times 10^2$ cells per well and incubated with 10% FBS for 2 weeks. After 2 weeks, clones were fixed with methanol and stained with Giemsa, and the number of colonies

was counted under a microscope.

**Wound-healing and invasion assays**

For the wound healing assay, an acellular area was created with a 10 μL pipette tube, and the extent of wound closure was observed after 0 and 48 h. Migration was quantified by measuring the distances that the cells migrated toward the original wound field.

For the invasion assay, a final concentration of 1×$10^5$ cells/mL was seeded into the upper chambers (coated in Matrigel) in serum-free medium. The lower chamber of the Transwell was filled with culture media containing 10% FBS as a chemoattractant. After the chambers were incubated at 37°C for 48 h, the successfully translocated cells were fixed with 10% formalin, stained with 0.1% crystal violet for 30 min and counted under a light microscope.

**In vivo tumorigenic and metastasis assays**

Four-week-old female BALB/c-nu/nu nude mice were obtained from the Laboratory Animal Center of Nanfang Hospital, Southern Medical University. The animal experiments were performed in strict accordance with the recommendations in the Guide for the Care and Use of Laboratory Animals of the National Institutes of Health. The protocol was approved by the Animal Ethics Committee of Nanfang Hospital, Southern Medical University. The animals were fed with an autoclaved laboratory rodent diet.

For in vivo tumorigenicity assay, a total of 5×$10^6$ SLC25A21-overexpressing-M5 or SLC25A21-overexpressing-Caco-2 cells, HCT116 cells with stable

SLC25A21 downregulation, and the corresponding control cells, were subcutaneously injected into the upper limbs of mice. The tumors were allowed to grow for 15 or 24 days before the mice were euthanized after anesthesia, and the tumors were dissected. The tumor volume was recorded every three days and calculated according to the following formula: 0.5 × length × $wildth^2$.

To develop the in vivo metastasis model, mice were injected intravenously via the lateral tail vein with $2 \times 10^6$ cells. After 4 weeks of monitoring, the mice were euthanized after anesthesia. The lungs were removed from the adjacent organs by dissection and fixed using 10% neutral-buffered formalin. Subsequently, consecutive tissue sections were obtained and stained with hematoxylin-eosin (H&E) to observe the metastatic nodules of the lungs under a microscope. The number of colonies was counted.

**IHC staining**

IHC staining was performed as described previously[2]. The slides were incubated overnight with anti-SLC25A21 (catalog DF4172, Affinity Biosciences; 1:80 dilution) or anti-Ki-67 (catalog GB111499, Servicebio, Wuhan, China; 1:200 dilution) antibodies at 4°C. After incubation with the secondary antibody at room temperature for 1 h, immunodetection was performed using diaminobenzidine (DAB) reagent (catalog K3468, Dako) according to the manufacture's protocol with a consistent reaction time for each section, and this step was followed by counterstaining with hematoxylin.

The IHC-stained sections were reviewed and scored separately in a double-

blinded manner. The staining intensity was scored as follows: 0 (no staining), 1 (weak staining), 2 (moderate staining) and 3 (strong staining=brown). The extent of staining, defined as the percentage of the positively stained areas in relation to the entire section, was scored on a scale of 0-4 as follows: 0% (0); 1%-25% (1); 26%-50% (2); 51%-75% (3); and 76%-100% (4). The summation of the staining-extent and staining-intensity scores was regarded as the final score for SLC25A21 (on a scale of 0-7).

**Mitochondrial isolation**

All experiments were performed using the Mitochondrial Isolation Kit for Cultured Cells (catalog 89874, Thermo Fisher Scientific) according to the instructions. Briefly, $2 \times 10^7$ cells were harvested, washed with PBS and centrifuged at 850 × g at 4°C for 2 min. Then, the pellet was resuspended in Mitochondria Isolation Reagent and incubated on ice for 2 min. Mitochondria Isolation Reagent B was added to the mixture, and the resulting mixture was ground and centrifuged at 700 × g for 10 min at 4°C. The supernatant was collected and centrifuged at 12,000 × g for 15 min at 4°C. The supernatant containing the cytosolic fraction was transferred to a new tube. The pellet containing the isolated mitochondria was resuspended in Mitochondria Isolation Reagent C and centrifuged again at 12,000 × g at 4°C for 5 min to obtain purified mitochondria for subsequent detection.

**Intracellular α-KG determination**

The levels of α-KG in the subcellular fraction were determined using the Alpha-

Ketoglutarate Colorimetric Assay Kit (catalog ab185440, Abcam) in strict accordance with the manufacturer’s protocol. In brief, $1\times10^6$ cells were homogenized in assay buffer and centrifuged to remove insoluble components. After the reaction, the absorbance of the microplate reader at 570 nm was read.

**Reactive oxygen species (ROS) production**

ROS were determined using the DCFDA-Cellular ROS Assay Kit (catalog ab113851, Abcam) according to the manufacturer’s instructions. Briefly, $1\times10^6$ cells were harvested and stained with 20 μM DCFDA at 37°C for 30 min in dark. After washing with 1X buffer, the relative ROS level was detected by flow cytometry with an excitation wavelength of 485 nm and an emission wavelength of 535 nm.

**$NADP^+$/NADPH assays**

The $NADP^+$/NADPH ratios were determined using the $NADP^+$/NADPH Assay Kit (catalog K347-100, BioVision, Milpitas, CA, USA) according to the manufacturer’s instructions. After cell lysis, the samples were mixed with the reagent provided in the kit, and the absorbance at 450 nm was corrected based on the blank controls. The concentrations of NADPH and $NADP^+$ were calculated using the NADPH standard.

**ATP measurement**

The ATP levels were measured with the ATP Colorimetric Assay Kit (catalog K354-100, BioVision). Briefly, $1\times10^6$ cells were lysed in assay buffer. The specific reaction was performed according to the manufacture’s instruction and

the absorbance was measured at 570 nm.

**Liquid chromatography-tandem mass spectrometry (LC-MS/MS) analysis**

The intracellular GTP levels were measured by LC-MS/MS. Briefly, 1 x $10^7$ of HCT116 cells with SLC25A21 depletion or control cells were scraped off plates in prechilled PBS and centrifuged to remove the supernatant, respectively. Each sample was added to 1 mL precooled methanol/acetonitrile/water (2:2:1, v/v) and shaken well. The mixture was ultrasonicated for 60 min in an ice bath, left overnight and centrifuged at 4°C for 30 min at 16,000 x g. The supernatant was collected, and the same amount of internal standard L-glutamate-d5 was added for vacuum drying (Eppendorf Concentrator plus). For mass spectrometry, 100 μL of acetonitrile-aqueous solution (1:1, v/w) was added, and the mixture was centrifuged at 16000 x g at 4°C for 20 min. The supernatant was collected and analyzed. Separation was performed by Nexera X2 LC-30AD (Shimadzu) high-performance liquid chromatography. The mobile phase consisted of the following: solution A was 5% acetonitrile solution and 10 mM ammonium acetate, pH 9; and solution B was 95% acetonitrile solution and 10 mM ammonium acetate, pH 9. The sample was dissolved in 100 μL of 50% acetonitrile solution in a 4°C autosampler at a column temperature of 40°C. The flow rate was 300 μL/min, and the sample size was 5 μL. A QTRAP5500 mass spectrometer (ABSCIEX) was used for mass spectrometry analysis in the positive/negative ion mode. The ion pairs to be tested were detected in the MRM mode. The chromatographic peak area and retention time were extracted

using MultiQuant software. The ion peak area of metabolite extraction was normalized by the internal standard L-glutamate-d5.

**Metabolism flux analysis**

For U-$^{13}C_5$-glutamine ([U-$^{13}C_5$]Gln) labeling, HCT116 cells with SLC25A21 depletion and control cells were respectively seeded at a concentration of 5 x $10^6$ cells. The day after seeding, the cells were rinsed twice with PBS before the addition of Gln-free RPMI 1640 medium supplemented with 2 mM [U-$^{13}C_5$]Gln (Cambridge Isotope Laboratories) and 10% FBS. After 2 h of incubation at 37°C, intracellular metabolites were extracted. The media were discarded, and the plates were placed on dry ice. The cells were washed three times with 4°C PBS and then incubated with prechilled methanol for 30 min at -80°C. Cells were scraped off plates in 80% methanol with 10 mM ammonium bicarbonate, and the mixture was processed via 5 cycles of 1 min of ultra-sonication at 1-min intervals in ice-water bath and incubated for 30 min at -20°C. After centrifugation at 15,000 × g for 15 min at 4°C, all supernatant was evaporated to dryness. The residues were reconstituted in 50 μL of 50% aqueous acetonitrile (1:1, v/v) containing 1 μg/mL phenylalanine-d5 prior to UHPLC-HRMS analysis. Chromatographic separation was performed using an Ultimate 3000 UHPLC system (Thermo Fisher Scientific) with a Waters BEH Amide column (2.1 mm × 100 mm, 1.7 μm). The eluents were analyzed with a Q Exactive™ Hybrid Quadrupole-Orbitrap™ mass spectrometry (Thermo Fisher Scientific) in heated electrospray ionization negative mode. The raw

mass spectral data were preprocessed by Xcalibur (version 4.0.27.19, Thermo Fisher Scientific; RRID: SCR_014593). IsoCor software was used to perform data correction by considering the contribution of natural isotopes.

**Determination of 50% inhibitory concentrations**

To establish the 50% inhibitory concentrations ($IC_{50}$) of CTX treatment, CRC cells were seeded onto 96-well plates (2 × $10^3$ cells/well) in 100 μL of culture medium overnight, and then treated with serial dilutions of CTX (Merck, Germany, a stock solution of 5 mg/mL). After 72 h of drug exposure, the OD value of different wells was recorded using a microplate reader to measure the $IC_{50}$ of the corresponding CRC cells.

**Methylation-specific PCR (MSP) assay**

Bisulfite conversion of the extracted genomic DNA was performed by using the EZ DNA Methylation Kit (Zymo Research Corporation) according to the manufacturer's protocol. Each PCR consisted of 20 ng of bisulfite-converted DNA, 0.3 μM primers, and 1 unit of KOD One™ PCR Master Mix (Toyobo) in a final volume of 10 μL. The following PCR cycle conditions were used: 98°C for 2 min, 35 cycles (98°C 10 s, 56.4°C 5 s and 68°C 15 s), and 68°C for 1 min. The amplified PCR products were separated by 2% agarose gel electrophoresis and visualized. The primers used for MSP are indicated in Supplemental Table S4.

**Dot blot assay**

The levels of 5-hmC marks in cells were detected by dot blot. Genomic DNA

was extracted from cells using the SteadyPure Universal Genomic DNA Extraction Kit (catalog AG21009, Accurate Biotechnology Changsha, China). After denaturation at 95°C for 5 min, DNA samples were spotted on nitrocellulose membranes at the indicated concentrations. After drying in a 60°C oven for 2 h, the DNA blot was exposed to ultraviolet light for 2 min, and incubated with anti-5hmC antibody (catalog 40000, Active Motif, Carlsbad, CA, USA; 1:10000 dilution) overnight at 4°C. After washing, the membrane was incubated with HRP-conjugated secondary antibody and visualized by enhanced chemiluminescence.

Table S1. The sequences of PCR primers for full-length human SLC25A21 and shRNA target SLC2A521

| Gene name | Sequence (5’-3’) |
|---|---|
| SLC25A21-F | CGGGGTACCATGTCCGCCAAGCCTGAAGTC |
| SLC25A21-R | GCTCTAGATCACCAGTTCTCTTGAAGCCATG |
| SLC25A21-shRNA | GGAGA GAUCA AGUAC AGAA |
| NC-shRNA | TTCTCCGAACGTGTCACGT |

Table S2. Sequences of primers used for qRT-PCR

| Gene name | Sequence (5’-3’) |
|---|---|
| SLC25A21-F | ATTTGGGATTGGTCTTCT |
| SLC25A21-R | GACTGTTGCCATTGTTTT |
| GAPDH-F | ACAGTCAGCCGCATCTTCTT |
| GAPDH-R | GACAAGCTTCCCGTTCTCAG |

Table S3. The sequences of siRNA

| Gene name | Sequence (5’-3’) |
|---|---|
| SUCLG2-siRNA | CUUGGAUAUUUCCAGAGAATT |
| NC-siRNA | UUCUCCGAACGUGUCACGUTT |
| SLC25A21-siRNA | GGAGAGAUCAAGUACAGAATT |

Table S4. The sequences of primers used for MSP

| Gene name | Sequence (5’-3’) |
|---|---|
| SLC25A21 M-F | TTGTTGTTTTATAGGGAGTTAACGC |
| SLC25A21 M-R | CTTCAAACTTAACGAACATCTTCG |
| SLC25A21 U-F | TGTTGTTTTATAGGGAGTTAATGTGT |
| SLC25A21 U-R | TTCAAACTTAACAAACATCTTCACC |
